# Tools for providing information to patients about high-tech medical resources for treatment of cancer

**DOI:** 10.2340/1651-226X.2026.45529

**Published:** 2026-10-02

**Authors:** Julie Gehl, Tatiana Michel, Erik Rokkones, Soleakhena Ken, Gregor Sersa, Irene Torres-Espallardo, Ester Orlandi, Laura Ridolfi, Nina Schmidt, Ingrid Kruecken, Barbara Leonardi, Margareta Haag, Aurélie Garcin, Anne-Laure Giraudet, Sandrine Lavallé, Colin Patrick Cantwell, Alexander Jorge Cortez, Chiara Marazzi, Roger Olofsson Bagge, Massimiliano Petrini, Francesca Bonifazi, Vincent Bourbonne, Frédéric Courbon, Ibrahim Edhemović, Lisa Licitra, Sergio Roman-Roman, Jean-Yves Blay, For complete list of author group, please see online version

**Affiliations:** a Department of Oncology and Palliative Care, Zealand University Hospital, Køge, Denmark; b Department of Clinical Medicine, Faculty of Health and Medical Sciences, University of Copenhagen, Copenhagen, Denmark; c Department of Luxembourg Research Clinic, Luxembourg Institute of Health, Strassen, Luxembourg; d Department of Surgical Oncology, Division of Cancer Medicine, Oslo University Hospital, Oslo, Norway; e Institut Universitaire du Cancer de Toulouse - Oncopole Claudius Regaud, Univ Toulouse France; f Department of Experimental Oncology, Institute of Oncology Ljubljana, Ljubljana, Slovenia; g Nuclear Medicine Department and Clinical Area of Medical Imaging, Hospital Universitario y Politécnico La Fe, Valencia, Spain; h Department of Clinical, Surgical, Diagnostic and Pediatric Sciences, University of Pavia, Pavia, Italy; i Radiation Oncology Unit, Clinical Department, National Center for Oncological Hadrontherapy (CNAO), Pavia, Italy; j Department of Medical Oncology, IRCCS Istituto Romagnolo per lo Studio dei Tumori “Dino Amadori” (IRST), Meldola (FC), Italy; k Patient & Public Involvement (PPI) Group, Luxembourg Institute of Health (LIH), Luxembourg, Luxembourg; l Board of Europa Donna Luxembourg asbl, Strassen, Luxembourg; m Board of Europa Donna – the European Breast Cancer Coalition, Milan, Italy; n ESMO PAWG (Patient Advocates Working Group), Lugano, Switzerland; o Myeloma Patients Europe, Carita Foundation Poland, Wrocław, Poland; p Swedish Network against Cancer, President, Stockholm, Sweden; q EUPATI Sweden, Board Member, Stockholm, Sweden; r Member of OECI Working Group for Patients, Rome, Italy; s Translational Research Department – SiRIC Curie, Institut Curie, Paris, France; t Centre Léon Bérard, Lyon, France; u St. Vincents University Hospital and University College Dublin, Elm Park, Dublin, Ireland; v Department of Biostatistics and Bioinformatics, Maria Sklodowska-Curie National Research Institute of Oncology Gliwice Branch, Gliwice, Poland; w Digital Medicine Center, Maria Sklodowska-Curie National Research Institute of Oncology, Gliwice Branch, Gliwice, Poland; x CNAO National Center for Oncological Hadrontherapy, Pavia, Italy; y Department of Surgery, Institute of Clinical Sciences, Sahlgrenska Academy, University of Gothenburg, Gothenburg, Sweden; z Department of Surgery, Sahlgrenska University Hospital, Gothenburg, Region Västra Götaland, Sweden; aa Experimental and Clinical Oncology of Immunotherapy and Rare Cancers, Immuno-Gene Therapy Factory, IRCCS Istituto Romagnolo per lo Studio dei Tumori “Dino Amadori” (IRST), Meldola (FC), Italy; bb IRCCS Azienda Ospedaliero-Universitaria di Bologna, Bologna, Italy; cc Service de Radiothérapie, Institut de Cancérologie et d’Imagerie, CHU Brest, France; dd INSERM, LaTIM, UMR 1101, Université de Bretagne Occidental, France; ee Institut Univ. Cancer Toulouse Oncopole, Toulouse, France; ff Department of Surgical Oncology, Institute of Oncology Ljubljana, Ljubljana, Slovenia; gg Faculty of Medicine Ljubljana, University of Ljubljana, Ljubljana, Slovenia; hh Medical Oncology 3 Department, Fondazione IRCCS Instituto Nazionale dei Tumori, Milano, Italy; ii Università degli Studi di Milano, Milano, Italy

**Keywords:** Patient navigation, nuclear medicine, radiomics, radiotherapy, surgery, cell- and tissue-based therapy, ablation techniques

## Abstract

**Background and purpose:**

High-tech medical resources play an increasingly important role in cancer diagnosis and treatment. However, accessing information about these technologies remains challenging due to their complexity and a lack of clear information pathways to obtain reliable information. This, in turn, contributes to inequalities in healthcare.

**Patient/material and methods:**

The Joint Action Network of Expertise on Cancer (JANE-2) is one of the largest networks under Europe’s Beating Cancer Plan. In JANE-2, the work package on High-Tech Medical Resources comprises seven domains of rapidly evolving technologies, namely Nuclear Medicine, Radiomics, Innovative Radiotherapy, Innovative Surgery, Physical Methods of Ablation, Cell Therapies, and Ex-vivo Testing of Agents. Leaders from these domains, together with patient representatives, have authored a review of the challenges of providing patients with information about high-tech medical resources, and have proposed potential solutions.

**Results:**

This article describes the challenges associated with providing adequate information to patients, the resulting consequences, and potential solutions. The article also specifically describes technologies related to the seven high-tech domains that are central to cancer treatment. Patient information tools related to these technologies are provided as supplementary material to the article.

**Interpretation:**

Cancer diagnosis and treatment are not only becoming increasingly effective but also more complex with the adoption of novel technologies. This article discusses the challenges and potential solutions related to the availability of patient information and provides examples of patient information tools for seven key and rapidly advancing technologies.

## Introduction

Technology in medicine is rapidly evolving and positively impacting cancer care with improvement of treatment outcomes and reduced side effects. However, for patients, it remains challenging to obtain a clear and comprehensive overview of these expanding technologies and their potential clinical relevance.

Local and national regulations provide extensive information on the availability of drugs for both physicians and patients. In contrast, information on technical resources in cancer treatment is often more difficult to obtain – both for patients and, to some extent, for treating physicians too. Furthermore, innovative technological solutions in cancer care are evolving rapidly, making it challenging to keep track of developments, to assess their relevance for individual cases, and to identify where such treatments are available.

Some novel diagnostic and treatment approaches are available only through clinical trials, limiting patient access both due to inclusion criteria and limitations in terms of which institutions are running the trial. Furthermore, patients who wish to stay informed of clinical trials need to access information through trial registries, if not offered at the institution that they are being treated. Although other new technologies may be available to patients, heterogeneity in cancer care means that access is not always guaranteed.

Information may be available through multiple channels, for example, at the hospital websites, scientific societies, and online searches (Figure 1). The quality of information depends on the source and may vary from high quality to downright misinformation. Understanding where information originates from and the level of evidence supporting it also poses a challenge.

**Figure 1:**
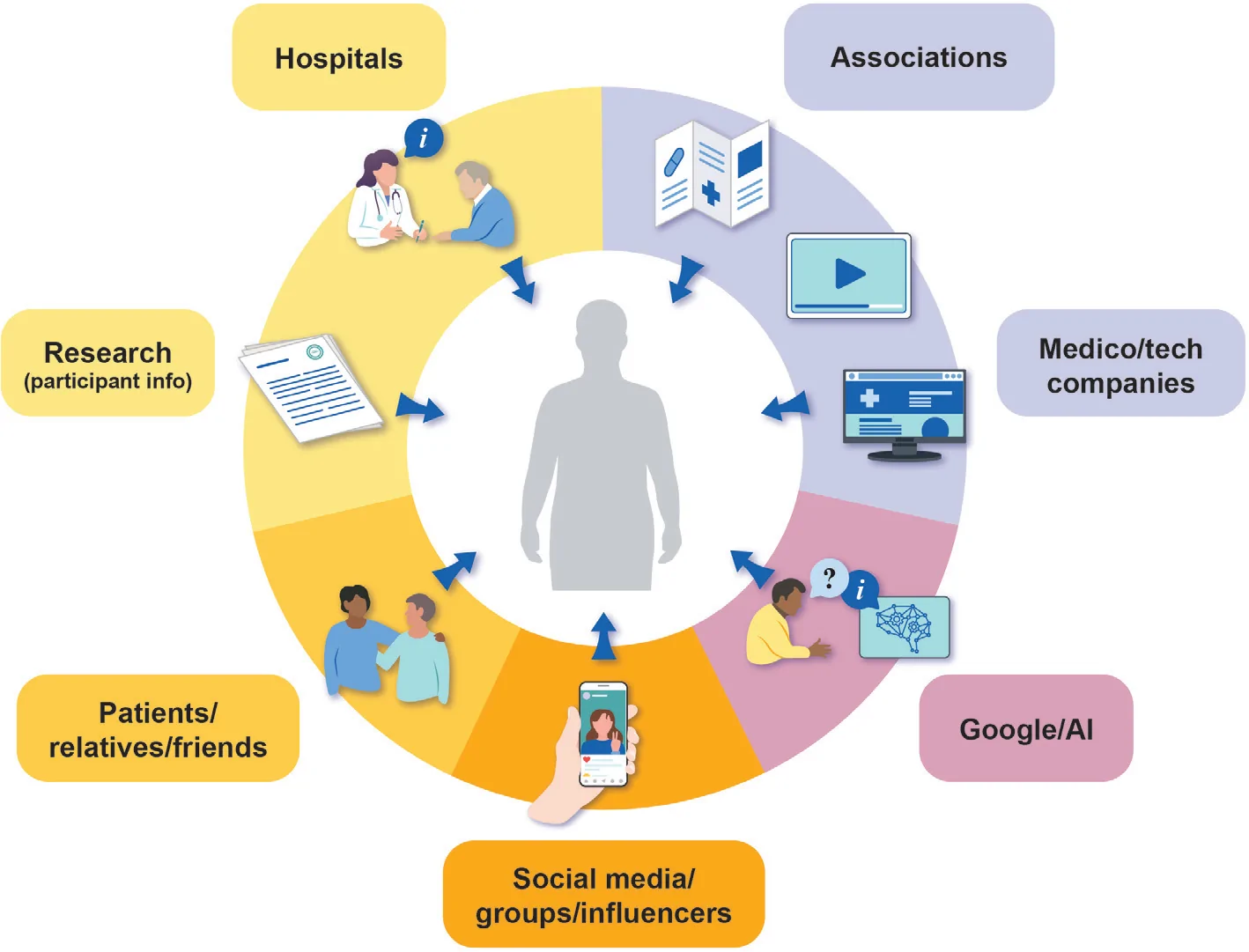
Patients obtain information from a variety of sources. Information may be given at the hospital before a procedure and/or in connection with participation in a clinical trial, but it may also be obtained from advocacy groups and organisations (e.g. patient organisations), industry (e.g. medico technical companies), search engines and/or AI, patient groups on social media, relatives, friends, care givers and fellow patients.

In addition to this, linguistic barriers, varying levels of health literacy, and differences in familiarity with technological developments may affect the understanding of the information provided.

Successful cancer care is based on a complex system of diagnostic technologies and medical interventions, carefully selected and planned for any individual patient. This is mainly done at multidisciplinary team meetings (MDTs) and by the treating clinicians. This requires that clinicians involved in MDTs are well informed about current technological developments, their availability, and their potential relevance for each patient.

The Joint Action Networks of Expertise on Cancer (JANE-2) aims to establish networks dedicated to improving cancer care at the European level through collaboration, and to identify topics in particular need of elevation. One such area is High-Tech Medical Resources, where seven networks of expertise have been established: Nuclear Medicine, Radiomics, Innovative Radiotherapy, Innovative Surgery, Physical Methods of Ablation, Cell Therapies, and Ex-vivo Testing of Agents.

For patients, the key practical questions often concern whether a given technology is relevant to their situation, whether it is locally available, and how it can be accessed. This article aims to support patients, relatives, caregivers, and decision makers by providing tools to facilitate access to information on high-tech medical resources.

This review outlines key aspects related to the provision and access to information about High-Tech Medical Resources, the associated challenges, and potential solutions. Up-to-date information sheets are included in the supplementary material to this article.

## Material and methods

This article has been prepared by authors representing the seven networks of expertise within the High-Tech Medical Resources work package under the JANE-2. Patient representatives have contributed both as authors of this article and as reference groups for the text in each of the networks of expertise. The tool is intended to be updateable, acknowledging that availability, indications, and referral mechanisms may change over time.

The technologies covered by each network are described next and in the supplementary material (see suppl. 1–7).

## Results

### Information tools and obstacles for information

The seven networks of expertise describe the complex technologies involved, as well as their applications, availabilities and challenges in providing information. Although the seven networks of expertise represent quite different technologies, the challenge of providing information to patients is rather similar. Tables 1 and 2 summarise main obstacles in providing information to patients about these technologies, as well as clinical indications for use. These technologies have different levels of readiness, some are implemented and broadly available while others are mostly available through clinical trials. Technology readiness and access for patients are described in Table 3. In addition, the supplementary material for each of the networks is an information tool for patients.

**Table 1:** Main obstacles in providing information to patients about technologies.

	What are the main obstacles in providing information to patients about technologies?		
	Example 1	Example 2	Example 3
Nuclear Medicine	Fear due to radiation	Not aware about other medical disciplines	Few centres with the needed infrastructure
Radiomics	‘Invisible’ to patients	Imaging infrastructure availability	Research facility to perform radiomics analysis
Innovative Radiotherapies	Fast-moving and complex concepts linked to innovation, especially related to the mechanisms behind the treatment	Access needs to be facilitated by a specialist medical professional.	Ongoing generation of clinical evidence and patients’ expectations mismatch
Innovative surgery	Wide number of surgical procedures include combined and multimodal techniques designed for personalized surgery → difficult to navigate.	Old perceptions of surgery limit comprehension of new knowledge.	Different hospitals may use different procedures, making standardised information more difficult
Physical Methods of Ablation	Unclear referral pathways	The physician treating the patient may not be comprehensively aware of possibilities in ablation.	On the internet information is often technology specific, which does not give the full picture of options.
Cell Therapies	Complexity of the topic Difficult to explain ‘living drug’ concepts Difficult to explain the risk of insertional mutagenesis	Patients are not always aware that cell and gene therapy (CGT) options exist for their conditions. Varying regulatory frameworks across regions make it difficult to provide consistent information on trial availability or approval status.	Some patients are not referred, or they are referred too late and therefore never receive treatment.
Ex vivo Testing of Agents	Several technologies are in research stage → not at the standard of care level	Ex vivo are not yet standard therapies, making general patient information more difficult. Several clinical trials have been conducted or are ongoing to address the feasibility and clinical validity.	Ex vivo technologies are diverse and complex to explain.

**Table 2:** Clinical indication for which technologies are relevant.

Diagnosis	Technology						
	Nuclear medicine	Radiomics	Innovative radio-therapy	Innovative surgery	Ablation	Cell therapies	Ex-vivo testing
Breast Cancer		X	X	X	X	Not yet, under evaluation	X
Central Nervous System Cancers		X	X	X	X	X	X
Gastro-intestinal Cancer	X	X	X	X	X	Not yet, under evaluation	X
Head and Neck Cancer		X	X	X	X	Not yet, under evaluation	X
Hematological Cancers	Not yet, under evaluation [1]	X	X			EMA and FDA Approved products	X
Lung Cancer		X	X	X	X	Not yet, under evaluation	X
Prostate Cancer	X	X	X	X	X	Not yet, under evaluation	X
Sarcoma		X	X	X	X	Yes for synovial Sarcoma	X
Skin Cancer		X	X	X	X	Yes, one FDA approved product for Melanoma	X
Urogenital Cancer	X	X	X	X	X	Not yet, under evaluation	X

EMA: European Medicines Agency; FDA: U.S.Food and Drug Administration.

**Table 3:** Technology readiness level and patient access.

Technology	Readiness Level	Patient Access/Limitations
Nuclear Medicine	Targeted radionuclide therapy (TRT) is not a single technology but a spectrum – ranging from fully commercialised, guideline-standard therapies to early-phase experimental agents. The most mature agents are EMA-approved and actively treating patients: Lutathera (¹⁷⁷Lu-DOTATATE) and Pluvicto (¹⁷⁷Lu-PSMA-617). Currently > 500 active clinical trials are underway, extending from early-phase academic trials to industry-sponsored, multicentre, multinational trials with both β and α particle-emitting radioisotopes.	Patient Access: Many patients face access barriers because of travel burden, cost, and other logistical constraints. Limitations: - Workforce. The lack of a suitably trained workforce is a major limiting factor. - Access & equity. Different reimbursement situation at national level across Europe can be found, hindering the availability for all European citizens. - Radioisotope supply. Production and distribution infrastructure for isotopes like ¹⁷⁷Lu is still scaling up globally.
Radiomics	Although evidence level is high, readiness of the radiomics technology is still limited to the specific area of research despite scientific community efforts for standardisation and harmonisation processes.	Radiomics is available across Europe but upon imaging infrastructure. Imaging data are exploited in a very heterogeneous manner and patients may not be aware that their imaging data are used for radiomics analysis.
Innovative Radiotherapy	Highly variable for the most innovative resources. Full technology readiness for most of them.	Unequal access across Europe due to limited availability of the innovative technology in specialised centres, high costs, variable reimbursement, and still-evolving evidence for some indications.
Innovative Surgery	Surgery is available across Europe. For many innovative approaches evidence level is low and more data are needed for readiness.	Access is heterogeneous for several reasons. Most important is a lack of standardisation of care structures and therefore surgeons with necessary skills, a lack of evidence, and a lack of investment in equipment.
Physical Ablation	Physical Ablation technology is licenced and commercially available worldwide. Image guided lung, liver and renal tumour ablation by interventional radiologists are well established in many European countries but not in all. There are continuous efforts to expand utilisation, improve primary efficacy and to quality assure practice of different technologies and techniques to treat tumours in different anatomical locations.	There is inequity of access to physical ablation across regions and European nations. Limitations include a lack of: - patient information about possibilities - access to trained personnel and equipment - insurance and health service recognition of the treatment - appropriate funding - systematic integration in care paths - guidelines - involvement of trained specialists at multidisciplinary patient conferences.
Cell Therapies	Seven CAR‐T products have received centralised marketing authorization by the European Commission (EC) covering 15 haematologic malignancies. Custom-made CAR-T therapies are provided under hospital exemption framework in some European countries. High readiness for approved CAR T cell therapies in haematological malignancies; medium readiness for emerging indications; low readiness for most solid tumour applications	High costs cause disparities in CAR-T therapy access among European countries. CAR-T therapy should be administered in highly specialised accredited centres. Access restricted to a limited number of accredited centres, creating geographical inequalities across Europe. Most cell-based therapies for solid tumours remain available only through clinical trials. No centralised European platform exists to identify accredited centres providing cell therapy treatments. Delayed or missing referral prevents some eligible patients from receiving treatment Patient information is fragmented and individuals may rely on informal or non-validated sources
Ex vivo Testing	Trials in progress; validation of technology effectiveness	Limited access for patients due to limited number clinical trials; Few selected hospitals available

### Nuclear medicine network of expertise

Nuclear medicine is a well-established medical discipline for diagnosis and treatment of several diseases using radiopharmaceuticals (Figure 2). A radiopharmaceutical is a medical compound that contains a radionuclide, the radioactive isotope. In the recent years, new and very specific molecules have been developed that bind almost exclusively to cancer cells, providing a specific method to effectively treat lesions when using therapeutic radionuclide (alpha or electron emitter). This leads to a significant increase in the applications of targeted radionuclide therapy (TRT) across cancer types [2–4]. Although TRT works across different cancer diagnoses, the technique is still specific to cellular targets that may limit application. Target expression, for example, prostate-specific membrane antigen (PSMA), must be confirmed by imaging before treatment. Heterogeneous expression means some lesions may be missed or undertreated. Although toxicities are low, off-target radiation to kidneys, salivary glands, and bone marrow can limit the number of treatment cycles and cumulative doses.

**Figure 2:**
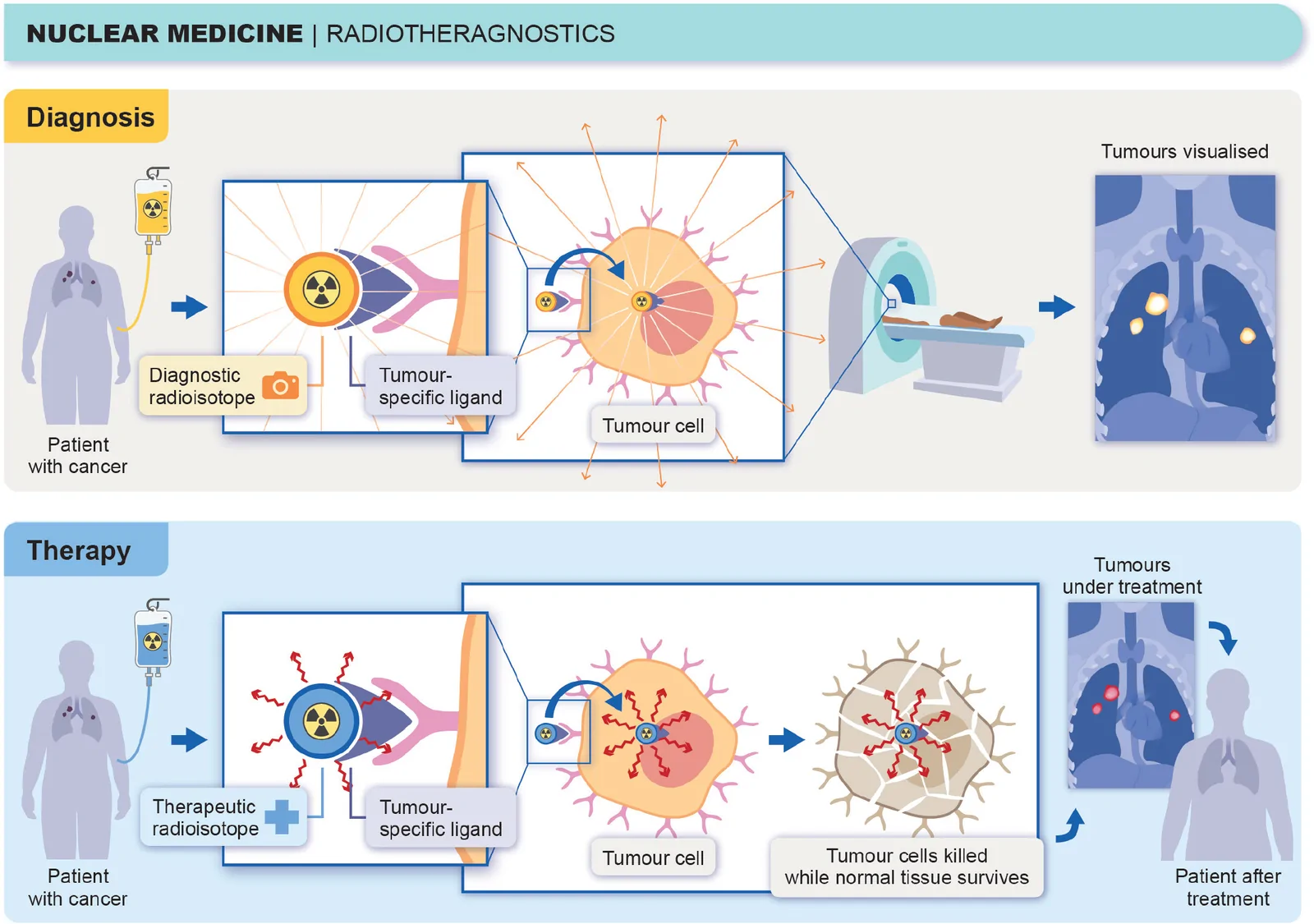
Radiotheragnostics uses a single tumour-targeting ligand paired with two different radioisotopes: one for imaging, one for therapy. In both steps, the ligand is radiolabelled – chemically linked to the radioisotope – and then administered intravenously. The radioligand circulates and binds specifically to its target on the tumour cell surface, where it can be internalised. In the diagnostic step, the radioisotope emits photons (or positrons) that pass out of the body and are detected by SPECT or PET, visualising the tumour and confirming that the target is present. In the therapeutic step, the same ligand carries an isotope emitting particles (β^-^ or α) that deposit their energy over a short range within the tumour, causing DNA damage and cell death while largely sparing surrounding tissue.

For the time being, this modality of treatment is an option when the type of cancer is resistant or unresponsive to other treatment lines. TRT is well tolerated and causes fewer side effects.

The use of these radiopharmaceuticals has been included in standard care through clinical trials that have demonstrated an increase in treatment effectiveness, overall survival and quality of life. With a theranostic approach, imaging of the lesions in the diagnostic phase predicts treatment response as the same molecule will be used for treatment. Therefore, this theranostic approach combines therapy and diagnostics, leading to a highly personalised clinical pathway for patients whose cancer disease is targetable by these ligands.

A highly qualified and MDT is needed for TRT administrations, including nuclear medicine physicians, medical physicist, radiopharmacist in close collaboration with oncologists and other referring physicians. This requires deep understanding of the target diseases, pathology, pharmacology, technical expertise in safe handling of the radiopharmaceuticals, knowledge in dosimetry and radiation safety and greater engagement with patient management [5]. The centres delivering TRTs must be authorised to manipulate radiopharmaceuticals and to use imaging devices (such as positron emission tomography [PET]/computed tomography [CT], PET/magnetic resonance [MR] and Single-Photon Emission Computerized Tomography [SPECT]/CT). The production of the radionuclides takes place in nuclear reactors, cyclotrons, linear accelerators and generators in highly specialised sites.

The administration of the radiopharmaceutical takes approximately 30 to 60 min. Since radiation is being used for the treatment, the patient is informed before and after treatment regarding radiation protection recommendation that must be followed to minimise the exposure of their family, relatives and friends. Depending on national regulations on radiation protection and the specific radionuclide being used, the patient may have to stay in a protected room for some days. Some new treatments, such as Lu-177 and alpha particle emitters, are characterised by low radiation levels outside the patient, allowing outpatient treatments. As the field of nuclear medicine evolves, several initiatives have been raised to provide information to patients.

For information for patients, see Suppl. 1.

### Radiomics network of expertise

Radiomics is a rapidly growing research field that combines medical imaging with advanced quantitative analysis. Its main purpose is to look at hidden information in medical images, that cannot be perceived by the human eye. Radiomics achieves this through the extraction of numerical features, which are then integrated into models for diagnostic, prognostic or predictive purposes. The key steps of radiomics are as follows (Figure 3):

**Figure 3:**
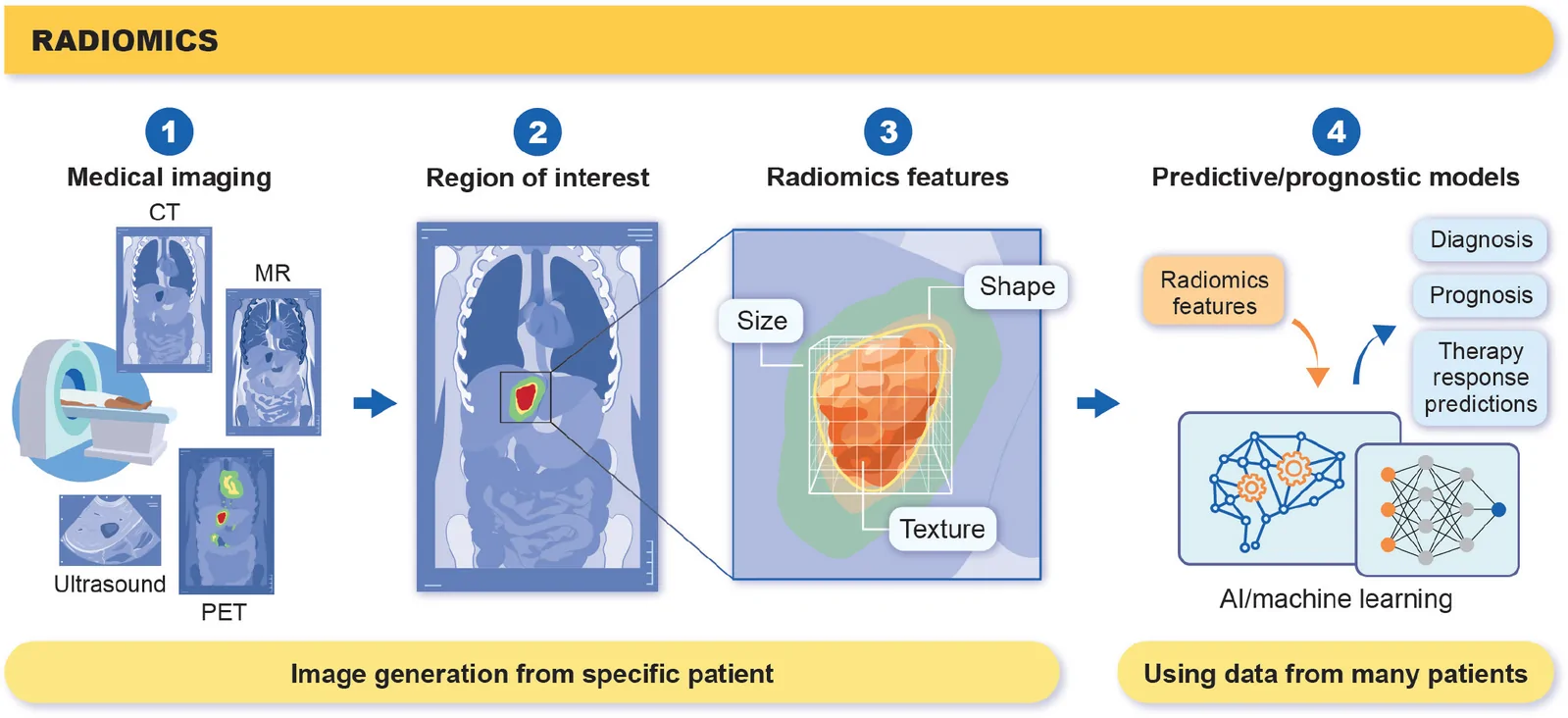
Typical steps in a radiomics analysis workflow: 1) Image/scan acquisition from different medical imaging modalities, either computed tomography (CT), magnetic resonance imaging (MRI), positron emission tomography (PET) or ultrasound. 2) Focus on the region of interest (e.g. the tumour). 3) Extraction of features from the region of interest like characteristics of size, shape or texture. 4) Integration of numerous radiomic features, extracted from multiple patients, into AI/machine learning models to make prognosis or predictions.

- *Tumour segmentation:* In medical images, characteristics of relevant regions, like tumour sites, can undergo a radiomics analysis. Therefore, the delineation of regions of interest is a crucial step.
- *Image preprocessing:*The quality of images varies as they are acquired at different times, and from different equipment and parameters settings. Preprocessing and harmonisation of images is required to conduct proper radiomics features extraction.
- *Features extraction:* Several 100 features are computed from the medical images. These features are known as radiomics features and belong to different categories, from simple categories such as intensity-based or shape-based to more complex features such as texture-based.
- *Models definition:* Radiomics’ features are analysed with statistical methods, but machine-learning algorithms can also be trained to aid in diagnosis, prognosis or decision of personalised-treatment [6].

Radiomics can be performed at any hospital equipped with medical imaging technologies, such as CT, MR, PET, x-rays or ultrasound. It may be relevant for any patient whose treatment involves imaging. While the technology has broad applications, it is particularly valuable for defining margins in radiotherapy and surgery.

Radiomics has high potential for clinical application. Standardisation and harmonisation in radiomics already exist thanks to efforts by the scientific community which shares extraction algorithms and image processing workflows [7].

Wider participative studies will help to share and compare results obtained from different centres and practices. Additional external and independent studies are needed to validate radiomics models, before their integration into clinical trials and diffusion in clinical routine [8].

For information for patients, see Suppl. 2.

### Innovative radiotherapies network of expertise

More than 50% of patients with cancer receive radiotherapy during their treatment. Ionising radiation damages the tumour cells’ DNA, preventing growth and replication as well as leading to tumour cell death. Radiotherapy is primarily delivered via linear accelerators (LINACs) by MDTs and is used alone or alongside other cancer treatment approaches. The primary objective is to irradiate the tumour target while sparing healthy tissues to maintain the best outcome in terms of cure and long-term quality of life. Innovative radiotherapy (iRT) utilises cutting-edge technology for pinpoint accuracy. Stereotactic radiotherapy (SRT/SBRT) delivers high, surgical-like doses in significantly fewer sessions than traditional methods. To ensure precision in delivery, image-guided radiotherapy (IGRT), adaptive therapy (i.e. adapting the beam delivery during the treatment course) and motion management (e.g. synchronising the beam delivery with natural breathing) are used. Other technical improvements include hybrid machines for better tumour targeting and treatment adaptation (e.g. MR imaging with a LINAC, MR-LINAC). Another level of innovation is the use of hadrons that allow to exploit biological and physical properties of particles (e.g. protons and carbon ions) to improve the treatment of specific tumour types (Figure 4).

**Figure 4:**
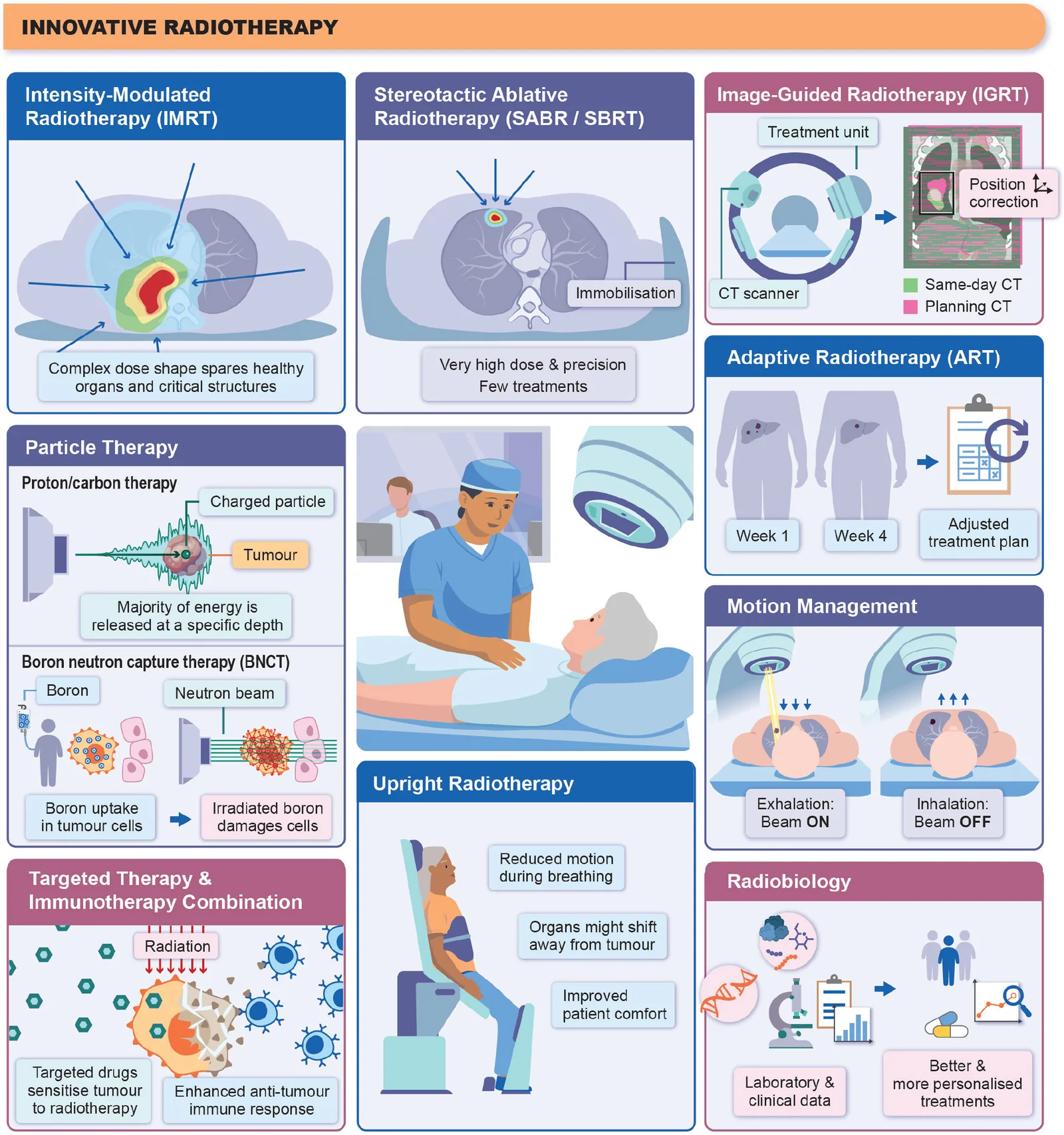
An overview of different types of innovative radiotherapy and related treatment approaches, highlighting how radiotherapy is becoming more precise and personalised, and limiting damages to healthy tissues. The most suitable technique depends on the type, size and location of the tumour, as well as the aim of treatment. Novel advances include continuous improvements in dose delivery (Intensity-Modulated Radiotherapy and Stereotactic Radiotherapy), higher precision (Image-Guided Radiotherapy, Adaptive Radiotherapy, Upright Radiotherapy and Motion Management), novel forms of radiotherapy (Particle Therapy), combination with medical treatments (e.g. Targeted Therapy and Immunotherapy), as well as continuously improved understanding of Radiobiology.

These specialised techniques are primarily indicated for complex and/or radioresistant tumours near vital organs, motion-sensitive sites (e.g. lung, liver, pancreas), paediatric patients, or complex re-irradiation. Furthermore, radiotherapy plays an increasingly important role in oligometastatic disease (metastatic disease with only one or few lesions, where treatment may be curative [9]). Widely distributed departments provide state-of-the-art care with LINACs, while specialised modalities, such as hadrontherapy, are centralised in specific centres. For such complex techniques, centralisation ensures the necessary multiprofessional expertise and furthermore addresses the high-cost infrastructure requirements [10–12].

Novel developments include automation of key radiotherapy processes (e.g. delineation and planning) with artificial intelligence (AI), the use of new radiation beams such as boron neutron capture therapy (BNCT) or ultra-high dose rate electron beams (FLASH), treatment in upright position for x-rays and/or hadrons. For some of them, their dissemination may necessitate the creation of referral networks to guarantee equal access to all European patients.

Innovative radiotherapy includes advanced technologies aimed at improving treatment precision and reducing radiation exposure to surrounding healthy tissues; however, the clinical benefit compared with standard approaches remains variable across tumour types and indications, and evidence is still limited for several emerging techniques. While approaches such as SBRT, MR-guided radiotherapy, and particle therapy are established in selected clinical scenarios, broader implementation is constrained by limited comparative evidence, high infrastructure costs, and unequal access across European healthcare systems.

For information for patients, see Suppl. 3.

### Innovative surgery network of expertise

Surgical oncology has progressively shifted from extensive open procedures to minimal invasive organ and function preserving approaches including robotic assisted procedures, through multimodal treatments emphasising both cure and quality of life. Advances in imaging, systemic therapy, and image guided surgery have enabled integrated pathways combining prehabilitation, chemotherapy, immunotherapy, targeted therapy, radiotherapy, and minimally invasive techniques, especially by robotic surgery platforms. However, this growing complexity challenges patient understanding and shared decision making.

Organ sparing cancer surgery integrates technical, pharmacologic, ablative, imaging, and radiotherapeutic components already established in modern care. We expect the development of new and innovative procedures in the coming years to expand towards novel treatments (Figure 5), including:

**Figure 5:**
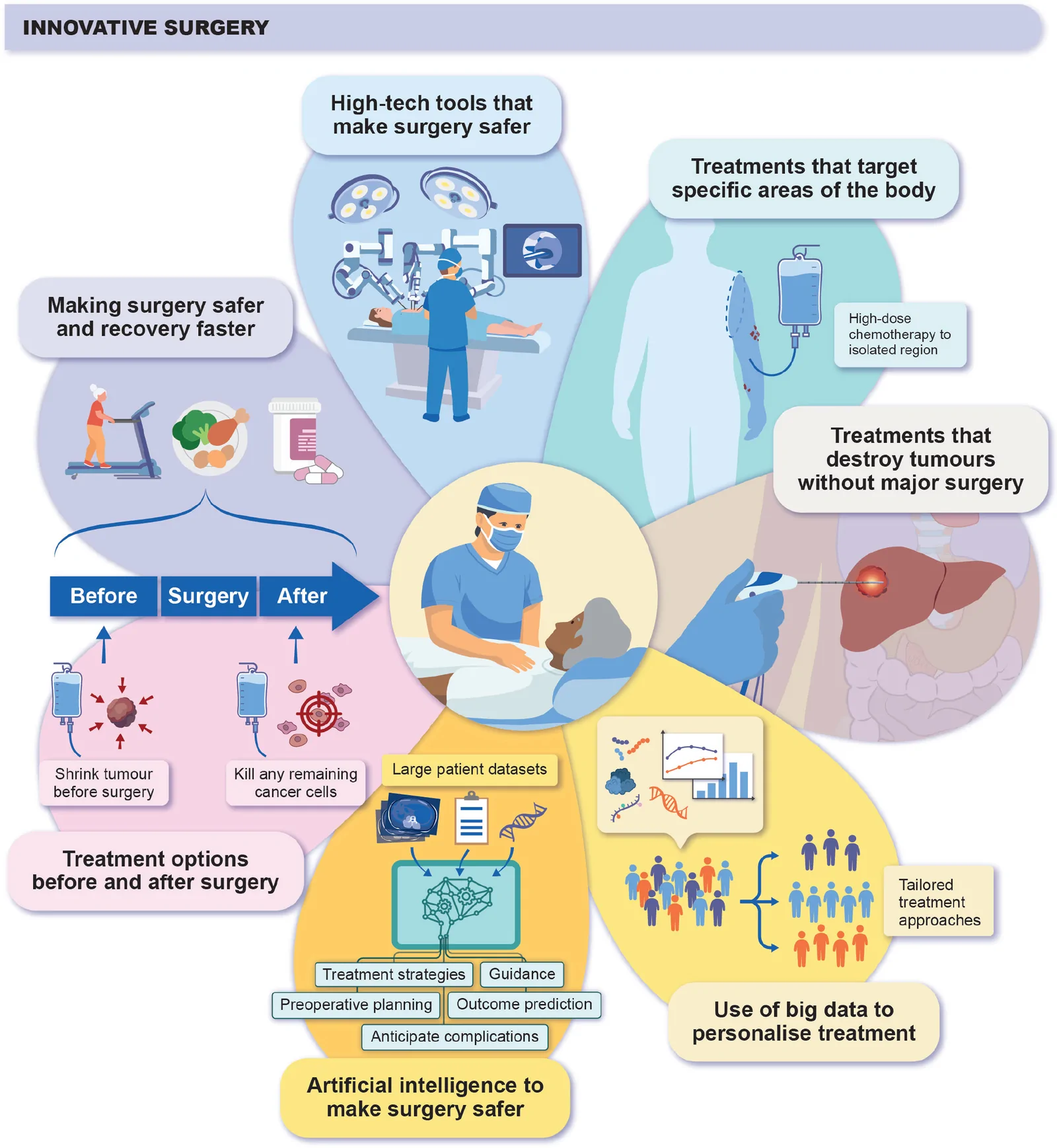
Surgical treatment may apply to all solid cancers. Innovative strategies include combinations with supplementary procedures, for example, medical treatment, radiation or ablation to increase efficiency and preserve normal tissue and function. Other examples include use of artificial intelligence and big data to make surgery safer and more personalised. It may also include perioperative physiotherapy and nutrition, helping patients tolerate the treatment better, and use of high-tech tools such as advanced imaging platforms to limit the operation and preserve healthy tissue and organs.

- *Enhanced perioperative management:* Prehabilitation and ERAS (enhanced recovery after surgery) programs increasingly support, for example, surgery for gastrointestinal and gynaecological malignancies [13].
- *New neoadjuvant and adjuvant strategies* (systemic medical antitumour treatment prior to or after surgery to enhance success rate): Immunotherapy and targeted combinations are now standard for multiple tumours (skin, lung, colorectal, urogenital), enabling improved likelihood of adequate complete tumour resections, organ preservation or even avoiding surgery [14–16].
- *Robotic surgery and advanced navigation and visualisation:* Robotic surgery, integration of image guidance, intraoperative diagnostics, fluorescence imaging, and 3D modelling (e.g. BrainLab, intraoperative MR, 3D heart/lung modelling) are expanding [17–19].
- *Localised and regional therapies:* These include isolated perfusion, HIPEC (hyperthermic intraperitoneal chemotherapy), PIPAC (pressurised intraperitoneal aerosol chemotherapy), and liver directed treatments [20, 21].
- *Ablative techniques:* For example, HIFU (high-intensity focused ultrasound) for prostate tumours and radiofrequency or microwave ablation of tumours in the liver can reduce the need for radical surgery [22].
- *Artificial intelligence:* Increasingly supports planning, guidance, and outcome prediction [23].
- *Multiomics and big data integration:* Combining molecular and imaging analytics; AI tools now aid patient selection (e.g. colorectal cancer ‘Florence’) and enhance robotic surgery with real time guidance and individualised training [24].

Side effects to surgery are both immediate complications (e.g. haemorrhage and infection), as well as long term sequelae such as impaired organ function, postoperative pain, altered body appearance. The novel techniques aim to reduce side effects and complications. Side effects and sequelae are highly dependent on type of surgery, indication and complexity of the procedure. An issue for patients can be that it is not always easy to anticipate the consequences of planned surgery, which can make the decision-making difficult.

For information for patients, see Suppl. 4.

### Physical methods of ablation network of expertise

Physical methods of tumour ablation are therapeutic approaches for elimination of local tumours, generally by once-only treatment [25]. These interventional therapies comprise thermal methods that use radiofrequency waves (RFA) [26] or microwaves (MWA) [27] to destroy tumour tissue by generating heat, and conversely cryotherapy [28] uses extreme cooling to cause tissue destruction. Non-thermal methods, such as electroporation (including electrochemotherapy) [29] or high-intensity focused ultrasound [30], do not rely on heat but, respectively, on cell permeabilisation using electric pulses or cell disruption using ultrasound. These methods are increasing in precision and applicability across different organs in the body (Figure 6). Physical methods of tumour ablation are effective across tumour types, including primary and secondary tumours, oligometastatic disease, and for providing symptomatic relief. Ablation can only be used in localised tumours, although more than one tumour may be treated.

**Figure 6:**
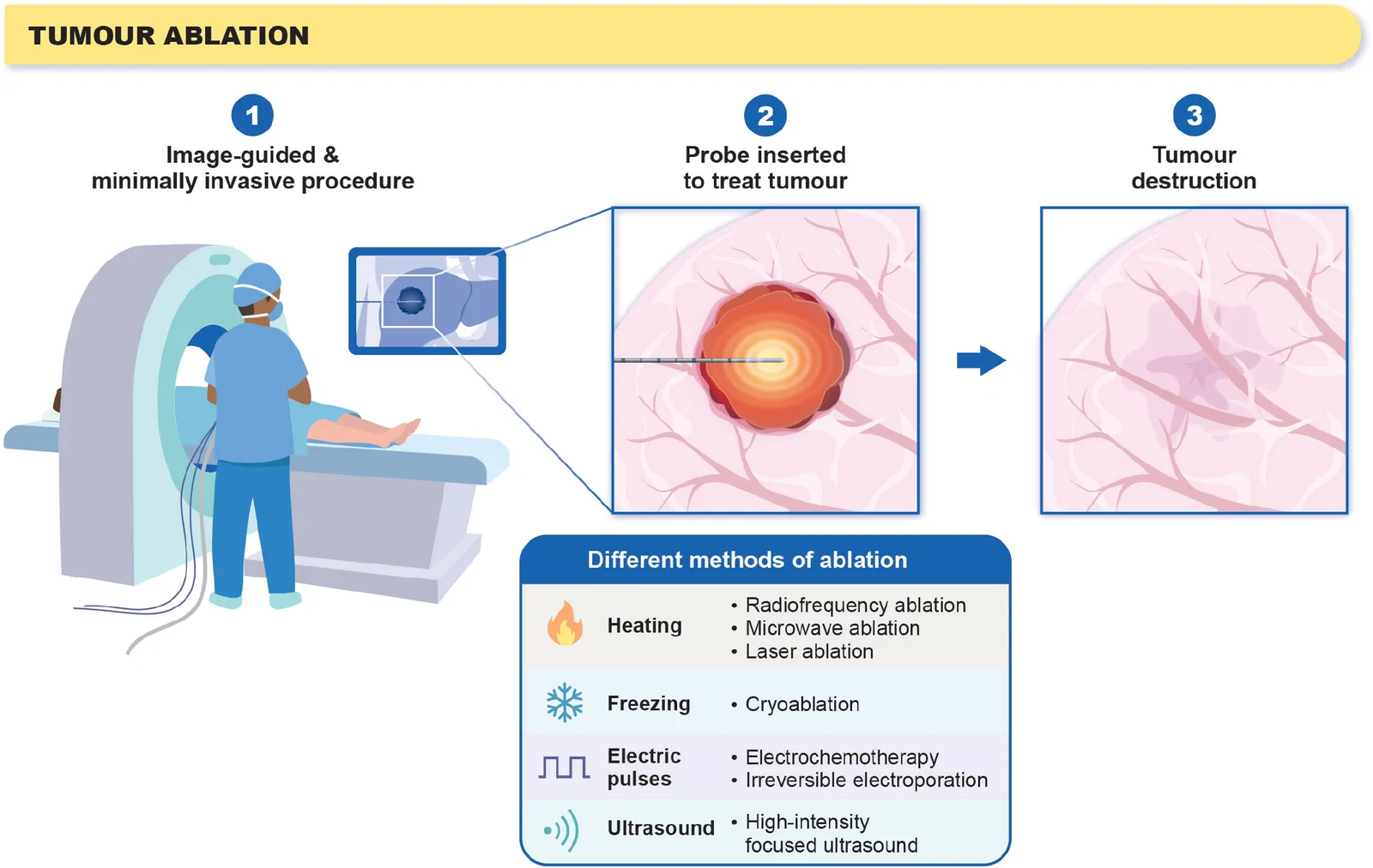
Ablation methods work across cancer types, exemplified here with a liver tumour and microwave ablation. The tumour is first visualised, and a thin needle (probe) is then inserted into the tumour and turned on, causing tumour destruction. The technologies used employ either heating, freezing, electric pulses or ultrasound. Ablation is highly efficient on localised tumours and typically only one treatment is needed.

Physical ablation therapies are available to cancer patients in oncology centres with comprehensive facilities. Not all methodological approaches are offered in every hospital; patients can obtain more information about the availability of these technologies through information at hospitals.

Patients are referred for treatment with physical ablation therapies through a MDT meeting, usually by their medical oncologist, surgeon or radiologist. Therefore, it is important that technologies and their effectiveness are widely disseminated and explained to increase accessibility of this treatment approach within the broader community. In this regard, patient societies may play an important role in explaining the potential of ablative therapies.

Physical ablation techniques are increasingly integrated into clinical practice, also in combination with systemic therapies. The technologies are maturing at different paces, thus RFA and MWA has a long-standing record, in particular for treatment of liver tumours [26, 31], whereas other technologies such as cryotherapy are evolving and are being used with new indications such as treatment of metastatic lymph nodes, and electrochemotherapy for cutaneous metastases. Due to the differences in potential efficacy and side effects of various technologies in different disease manifestations, collection of data via international collaborations are essential [29].

The rapidly evolving treatment landscape poses challenges for patients seeking information regarding outcomes and side effects. Furthermore, ablation depends very much on the tumour size, anatomic location, as well as proximity to vital normal tissue structures. This makes personalised treatment plans necessary, and makes it more difficult for non-experts to know if the treatment is a possibility.

For information for patients, see Suppl. 5.

### Cell therapies network of expertise

Cell therapies are classified as Advanced Therapy Medicinal Products (ATMPs) and include both somatic cell therapies and genetically modified cell therapies. They comprise cells or cell‑based preparations that have undergone substantial manipulation to alter their biological characteristics, physiological functions, or structural properties. The cells used may be autologous (derived from the same patient), allogeneic (from a human donor), or xenogeneic (from a non-human donor) [32]. Please see Figure 7.

**Figure 7:**
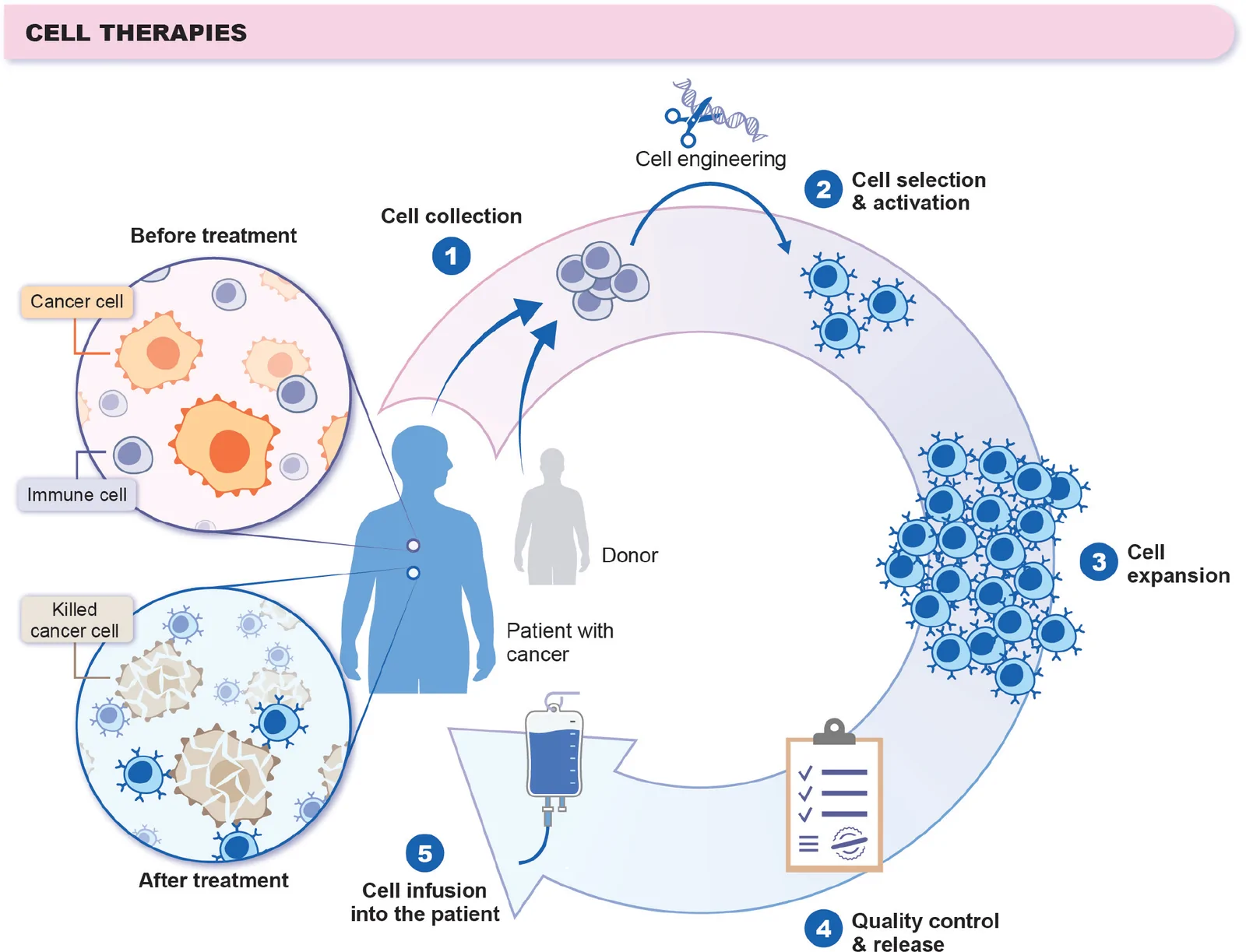
The concept of cell therapies. 1) Cells are collected from healthy donors (allogeneic) or from patient (autologous). 2) Target immune cells are isolated, activated with specific stimuli and in some cases genetically engineered to enhance their therapeutic function, including CAR insertion. 3) The cells are grown and increased in number in a good manufacturing practice (GMP) laboratory before being used for treatment. 4) The cell product undergoes extensive safety and quality testing. 5) The final product may be infused to the patient.

Genetically modified cell therapies represent a subset of immunotherapeutic interventions in which immune effector cells are isolated, expanded, and engineered ex vivo to enhance their capacity to recognise and eliminate pathological cells.

Through advanced manufacturing and engineering processes, these cells can be optimised to target diseased or abnormal cells, including cancer cells. Cell-based gene therapies involve the genetic modification of patient-derived or donor cells before reinfusion.

Access to cell therapies for patients with haematological or solid tumours is still restricted because only a limited number of specialised, accredited centres are authorised to deliver these treatments. These centres must meet stringent requirements for safety, quality, and clinical expertise, consequently limiting their number [33].

In addition, access is often further constrained by the scarcity of academic or non-profit clinical studies. Many patients can only receive treatment through commercially available ATMPs, and in several European countries the high cost of these products creates a major barrier to equitable access [34, 35].

At present, patients often identify these centres through informal channels such as social media, patient communities, or general internet searches, rather than through verified sources. A centralised European platform listing accredited hospitals providing cell therapy treatments for specific indications does not yet exist. This contributes to different access and potential delays in referral to appropriate clinical centres.

Currently, most cell-based gene therapies available in clinical practice are CAR‑T cell therapies, primarily indicated for haematological malignancies. Nevertheless, solid tumours with a strong immunogenic profile such as melanoma, renal cell carcinoma, and non–small cell lung cancer, may also benefit from cell-based therapeutic approaches. An example is the cell therapy lifileucel that was approved by the U.S. Food and Drug Administration (FDA) for treatment of adult patients with unresectable or metastatic melanoma [36]. As the field evolves, transparent, accessible, and harmonised information on accredited treatment centres will be essential to ensure equitable access across Europe, including emerging non-oncological indications such as autoimmune disorders.

For information for patients, see Suppl. 6.

### Ex vivo testing of agents network of expertise

Cancer treatments are often selected based on standard clinical guidelines and genetic testing. While this approach benefits many patients, it does not always identify the most effective treatment for the individual patient.

Ex vivo testing offers a complementary approach currently under development in laboratories [37]. It allows researchers and clinicians to study how a patient’s own cancer cells respond to different treatments outside the body, supporting more personalised cancer care.

Tumour tissue collected during a biopsy or surgery is used to create patient-derived cancer models that keep tumour cells alive in the laboratory for testing. Rather than focusing only on genetic alterations, these models enable direct testing of multiple drugs or combinations to observe which treatments are the most effective.

Several ex vivo technologies are being explored (Figure 8), including:

**Figure 8:**
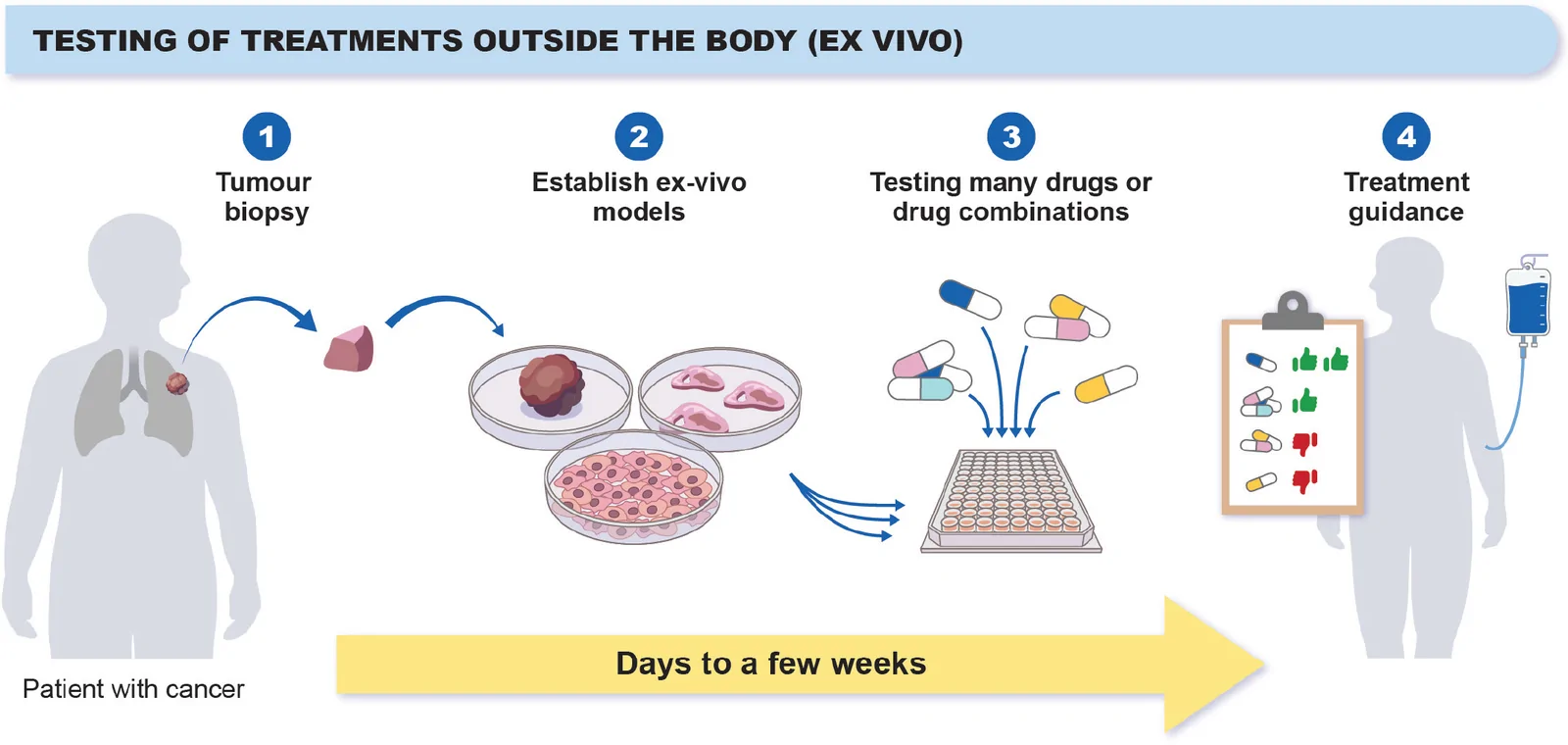
An overview of ex vivo drug testing and how it may be integrated into clinical care. A patient’s tumour sample is collected at diagnosis and/or at relapse as part of routine clinical procedures. Tumour cells are then processed in the laboratory using an ex vivo drug sensitivity screening platform. Multiple drugs are tested in parallel, which makes it possible to evaluate potential treatments. The results are then discussed by a multidisciplinary molecular tumour board, often together with genomic profiling data, to help inform individualised treatment decisions.

- *Patient-derived cancer cell lines*: Cancer cells grown on a flat surface, easy to study but may not capture tumour complexity.
- *Tumour tissue slices:* Thin pieces of fresh tumour, preserve the original structure for short-term testing.
- *Patient-derived xenografts (PDX):* Tumour tissue implanted into mice to better preserve tumour diversity.
- *Patient-derived organoids and assembloids*: Three-dimensional mini-tumours resemble patient tumours.
- *Tumour-on-chip systems*: Microfluidic devices recreate key features of the tumour environment, such as cell interaction and fluid flow.

These functional testing approaches aim to guide personalised treatment decisions based on how an individual patient’s tumour behaves, rather than relying solely on standard guidelines or genomic profiling [38]. Potential benefits include a more rapid identification and initiation of effective treatment, avoiding unnecessary treatments and side effects, and identifying alternative or off-label therapies, particularly for rare cancers or when standard treatments fail.

Despite encouraging progress, challenges persist, including accurately reproducing the tumour microenvironment, maintaining long-term cell cultures, and ensuring reproducibility. These methods are not yet part of standard clinical care across Europe. However, multiple academic-led clinical trials are currently evaluating the feasibility and clinical value of these approaches across different cancer types [39]. A major barrier to broader adoption of exvivo drug testing is the lack of standardisation. Although promising predictive value has been demonstrated, consensus is still needed on which ex vivo platforms should be prioritised. Clinical validation will rely mainly on co-clinical trials correlating ex vivo readouts (e.g. LD50, IC50, metabolic activity) with patient outcomes. Several such trials are ongoing, but ex vivo guided therapies remain investigational and are not yet available outside clinical studies.

For information for patients, see Suppl. 7.

## Discussion

### Advancing medical technologies

High-tech medical resources are increasingly used in cancer diagnosis and treatment, providing important advances in cancer care. In diagnostics, examples include the use of radiomics to characterise the extent of malignant lesions, and to access the biological characteristics and aggressiveness of tumours, thereby supporting treatment decisions. In another example, nuclear medicine is well known in the context of PET/CT scans using glucose uptake to show cancer extension, however several tumour-specific novel tracers and ligands can provide much more detailed information in several indications. In addition, nuclear medicine theranostics combines diagnostics of the tumour and its treatment, resulting in a more personalised therapy.

In cancer care, surgery, when possible, remains one of the first crucial steps, and is under constant development. Surgery broadly covers several sub-specialties and whereas some developments benefit across sub-specialties, others are tied to specific sub-specialties or surgical procedures.

Similarly, radiotherapy is advancing in all aspects, including improved target definition, sparing of normal tissues, and therapy delivery. Novel technologies, and their added value versus classical approaches, may not be easily available to all patients.

Ablation by physical methods, for example, radiofrequency, electroporation or cryotherapy is increasingly being used to ablate tumours that are either singular and/or resistant to other treatments or not eligible for resection by surgery, but their added value and limitations are not necessarily well described.

Cell therapies offer new options for cancers resistant to traditional treatments and continue to evolve with new technologies and new indications. Finally, ex vivo testing of agents uses biopsies from patients to test in laboratory systems, investigating whether drugs, including combinations of drugs, are likely to benefit patients, in essence establishing an avatar for the patient to test drugs for a specific cancer case.

Together, these novel technologies offer improvements and hope for broader groups of patients with cancer. These technologies also complement each other, for example, nuclear medicine informs radiomics, which in turn is applied in radiotherapy. Consequently, parallel developments may exponentially enhance patient outcomes.

### Availability of high-tech medical resources, and information to patients

The availability for individual patients is not uniform. Several factors contribute to inequity in options, and one of these is the lack of updated information about possibilities in diagnosis and treatment.

Patients with cancer have several possibilities to obtain information about cancer diagnosis and treatment. Information about technologies in cancer diagnosis and treatment is not available in a homogenous way, unlike information about cancer drugs or treatment guidelines, which is relatively uniformly available, and from central authorities. Patients may receive information from their cancer centre in connection with expected treatment through participant information in research protocols. This information is likely to be very accurate as experimental research protocols follow standards of care, however, the possibility to be included into these research protocols is not equally accessible for patients across Europe. Making this information available for patients in order to enable discussion with the treating physician and team is the topic of this article. It empowers patients. It may also facilitate access to innovation to a larger proportion of patients.

In many cases patient organisations have websites with extensive information about diagnostic and treatment options. However, this may differ from country to country, and although patients may be able to access information from sources outside their own country, this cannot automatically be assumed. Information from medico-technical companies is available online for patients but may not necessarily be balanced or suited for patients’ needs. Internet search engines and/or AI are increasingly used by patients and offer both well suited and easily understandable information, as well as unsuitable or even incorrect information. It can be difficult for patients to navigate. Patients are often linked to social media groups or are informed by posts, and are in contact with family members, friends and peer patients. Again, some information may be highly useful, and in other cases misplaced, incomplete or incorrect.

A survey examining additional information sources used by patients treated with surgical oncology [40] demonstrated that nearly 80% of patients accessed at least one non‑validated resource: 21% relied on friends and relatives, 20% on non‑governmental or hospital‑based resources, and 12% on social media platforms. Importantly, 23% of patients reported encountering conflicting information. According to the authors, these findings support the need to actively encourage patients to use validated information sources. By extension, this also calls for better information services by healthcare providers.

### Patient reported experiences and outcomes

Patient reported outcomes (PROs) and experiences (PREs) provide unique, patient centred nuances to clinical data and are highly relevant in the context of patient information and high-tech medical resources.

PROs and PREs are increasingly included in scientific literature, for example, in the form of questionnaires [41]. Semi-structured interviews further allow a broader dialogue about patient experiences and may be used to identify and quantify topics of general interest [42, 43]. In addition to these data on patient experiences and outcomes, there is a continuous exchange of patient experiences in online fora, as part of group activities and informal personal exchanges, see also Figure 1.

High-tech medical resources are continuously developing, in both jumps and increments. PROM and PREM reporting, if carried out systematically, can help to catch information about change in patient experience as the technologies develop.

### Technology readiness and patient access

The technologies described are at different readiness levels. While surgery and radiotherapy have been known for well over a 100 years, fields such as nuclear medicine, radiomics, physical ablation, cellular therapies and ex vivo testing of agents are younger. All fields are continuously developing, but at different stages of readiness. Some diagnostic and treatment opportunities are only available through clinical trials, limiting accessibility for patients.

It is particularly challenging for patients to navigate in a scenario where technologies are discussed, but not yet available, even in clinical trials. Some advances are available to select patients in clinical trials, whereas other technologies are available – but not necessarily to every patient due to heterogeneous distribution and bottlenecks in referrals.

Evidence for benefit and harms of novel treatment are generated in various ways. At times, a technology is updated without specific evidence for benefits and harms, and evidence may later be generated by comparing results before and after this change [44]. Evidence is often generated in smaller phase II trials, which may subsequently be combined in larger meta-analyses. Larger phase III studies allow a high level of evidence and have also been utilised. A number of international collaborations aim to collect large datasets for the study of results from treatment.

The pace at which technologies develop and become available, especially those that are not backed by large industry, can be painstakingly slow. Here, collaborative efforts are key to collecting and utilising data, in order to expedite evidence-based introduction into the clinic. This development is paralleled in, for example, personalised medicine where international collaboration can be necessary to generate evidence faster [45].

Table 3 shows technology readiness levels for the technologies, as well as level of patient access.

### Initiatives

Recently, a pan‑European initiative has been launched: the Berlin Institute of Health at Charité coordinates the project EU‑CiP (European Cancer Information Portal), funded through the EU Horizon Europe programme. EU‑CiP establishes, for the first time, a European network of patient‑centred, trustworthy, and evidence‑based cancer information portals. The initiative is a key component of the European Cancer Patient Digital Centre (ECPDC), developed under Europe’s Beating Cancer Plan and the EU Mission on Cancer. Patient information tools addressing these technologies should be aligned with and integrated into this European framework.

EUnetCCC is network focusing on development of compre-hensive cancer centres across Europe, which includes a strong focus on patient involvement and information to patients.

The JANE-2 network is part of Europe’s Beating Cancer Plan, the focus of which includes equal access to cancer diagnosis and treatment and improving quality of life for cancer patients and survivors. JANE-2 is a large collaborative project under which High-Tech Medical Resources as a topic is being advanced through increased collaboration and initiatives. This aims to provide better and more equal access to high-tech medical resources, and to understand how patients’ lives may be positively affected by increased knowledge of novel technological possibilities. One central element in this work is how patients may stay informed about relevant technologies.

### Do we need more patient materials or a change of strategy?

From the patient perspective, access to information about medical technology is not a secondary issue – it is a necessity for meaningful participation in one’s care. Patients want and need to understand the technologies used for their diagnosis and treatment. They ask concrete questions: How will this procedure affect my body? Is it safe? What are the short- and long-term side effects? How should I prepare? What will my daily life look like during and after treatment?

Despite the growing awareness around patient involvement in oncological care and shared decision-making, the information available to patients is often scattered, and difficult to understand. Sometimes, misconceptions and myths continue to permeate thoughts. For example, the term ‘radiation’ may evoke associations with contamination or long-term harm. Conversely, highly innovative methods, such as cell therapies, can be perceived as a miracle cure.

The challenge not only lies in the absence of information but also in the absence of information that is genuinely understandable and valid. Medical specialists, quite reasonably, communicate in a highly technical language, which may unintentionally create cognitive and emotional barriers for patients, whose levels of health literacy vary widely. Patient advocates, on the other hand, are well prepared to assess if the education material is clear, relevant, and respectful. However, they are rarely equipped with the methodological tools and skills needed to develop patient-friendly, scientifically robust materials in line with the plain language principles. As a result, both groups are often expected to fulfil a task that exceeds their core competencies and professional roles.

Currently, healthcare systems lack the structured involvement of communication professionals trained in plain language and patient-centred information design. These specialists can translate complex medical messages into accessible content without oversimplifying, infantilising, or compromising scientific accuracy. Integrating this expertise into oncology care would not require radical restructuring of healthcare systems but rather recognising that high-quality information and patient-friendly communication are components of holistic care.

If this need remains unaddressed and patients are left to fill information gaps on their own, they will most likely turn to the internet for answers. The risk is that widely used AI chatbots may become the ‘go to’ place and patients will receive information that is unverified, outdated, inaccurate, or misleading.

For patients to engage in shared decision-making, to consent for advanced procedures consciously, and to navigate increasingly complex patient journeys, we must systematically address the need to provide comprehensible information. The medical sector has reached a level of technological development that demands an equally advanced standard of communication. We need to shift our thinking from producing more materials for patients to designing deliberate, professional, and patient-centred communication. Otherwise, we risk leaving patients informed in theory but unsupported in practice.

### Strengths and limitations

Perhaps the greatest strength of this article is its very existence. Discussion about patient information is often absent from the scientific literature on cancer treatment, and this article helps to bridge the gap between the sphere of communication amongst experts and patients taking an interest in novel possibilities for treatment. It is also a strength of the article that seven diverse technologies are represented and that patient information tools are included as supplementary material.

The inclusion of patient representatives in the author group has led to important perspectives being considered and alterations being implemented.

A clear limitation is that this is a communication in only one language, whereas many patients do not readily access infor-mation in a second language, especially when this information is complex. This may be remedied by, subsequent to publication, translating patient information tools to more languages.

Another limitation is that it remains challenging for most experts to fully understand patients’ needs, and in particular, to communicate the key aspects of the technology with a level of detail that is relevant to each individual patient. Knowing this limitation, the author group has included patients’ representatives in the writing of this article.

## Conclusions

High-tech medical resources are developing rapidly and are becoming an increasingly important and integral part of cancer diagnosis and treatment. Staying informed about options is the wish of many patients, and it may also be important in health equality that patients have information about the technological possibilities. Validated information is sometimes difficult to access, and there is an abundance of information from unreliable or outdated sources.

Several actors have an important role to play here; medical and technical specialists have an important role to play in always factoring patient information into development of their field, also through scientific societies. National and European healthcare institutions should have strategies for providing precise, adequate and updated information to patients about technologies. Patient friendly versions of national guidelines should be considered. Furthermore, patient organisations already play an important role in providing information, but this could be further strengthened.

It is also important to keep medical communities informed so that every patient whose case is discussed at MDT conferences can be assured that the team has knowledge of all available treatment options.

Finally, as examples of patient information tools, the supplementary material in this article are written for patients and concern seven diverse and rapidly developing technologies: Nuclear Medicine, Radiomics, Innovative Radiotherapy, Innovative Surgery, Physical Methods of Ablation, Cell Therapies, and Ex-vivo Testing of Agents.

## Supplementary Material



## Data Availability

Not applicable.
