# Supplementary material for: Tools for providing information to patients about high-tech medical resources for treatment of cancer
Source: Acta Oncol. 2026 Oct 2;65:45529. doi: 10.2340/1651-226X.2026.45529 (PMC13635841; doi:10.2340/1651-226X.2026.45529)
Supplement: Supplementary file 1 [file AO-65-45529-s1.pdf]

Supplementary material has been published as submitted. It has not been copyedited, or typeset by Acta Oncologica

In connection with JANE-2, the following patient information sheets were developed:

- Patient information – Nuclear Medicine
- Patient information – Radiomics
- Patient information – Innovative Radiotherapies
- Patient information – Innovative Surgery
- Patient information – Physical Methods of Ablation
- Patient information – Cell Therapies
- Patient information – Ex-vivo Testing of Agents

By developing these information sheets, we aim to enhance information to patients about highly advanced technologies within cancer treatment.

The patient information sheets were developed by experts within each technology in collaboration with patient representatives.

The content of these information sheets may be downloaded, shared and translated to other languages. Referencing to this original material is required.

Supplementary S1: Patient information – Nuclear Medicine

Nuclear medicine is a medical area in which pharmaceuticals that emit radiation (radiopharmaceutical) are used for diagnosis and treatment by visualising molecular and functional changes.

Nuclear medicine therapy is a non-invasive treatment that offers people living with cancer a targeted and personalised care with minimal side effects.

Application of technology (what does it do)

A radiopharmaceutical is a combination of a *pharmaceutical* and a *radionuclide* (radioactive isotope) that emits radiation. These pharmaceuticals target a specific organ, tissue, or cellular receptor in the body. The radionuclide emits radiation in the form of photons that could be used for imaging by means of PET or SPECT devices (diagnosis), or in the form of electrons and alpha particles for treatment (therapy). The combination of diagnosis and therapy with the same molecule brings the word theranostic or radiotheranosis, a technology allowing to "see what you treat and treat what you see". The molecules used specifically seek out and stick to cancer cells. Once attached, radiation enters the cancer cell, damages its DNA and thereby stops the cancer cell from growing or cause it to die.

Who does it, where can you find it?

The diagnosis and the administration of therapy must be done in highly specialised departments within a hospital (Nuclear Medicine Departments) or dedicated centres with the corresponding authorisation to manipulate and administer radioactive sources, to assure safety conditions in the administration and reduce the radiation dose to the public.

The administration is performed by a nuclear medicine specialist who works in close collaboration with radiopharmacists, medical physicists, nurses, and medical technicians. The appropriate treatment is determined by the referring physician who may be an oncologist, endocrinologist, hepatologist or gynaecologist, depending on the type of tumour.

Information about departments or centres performing this procedure is provided by the referring physician. Furthermore, this information may be available on websites of the hospitals and dedicated centres.

Inpatient or outpatient?

The administration of the treatment is planned as an inpatient or outpatient procedure depending on the type of radionuclide, radiation dose and national safety regulations.

In the *outpatient treatment*, the patient is discharged the same day. It is used for lower radiation doses or short-lived isotopes (like lutetium-177 and radium-223).

Inpatient treatment is required for higher radiation doses or longer-lived isotopes. In these cases, it is necessary to stay in shielded hospital rooms until radiation levels are safe (e.g. treatments with iodine-131).

Upon discharge, a set of recommendations are provided to ensure minimal radiation dose to friends, family and caregivers in the following days.

There is a growing trend toward outpatient treatments as therapies become safer and more efficient.

Who may benefit?

Currently, this therapeutical option is being used in thyroid cancer, neuroendocrine tumours, prostate cancer, liver cancer (hepatocarcinoma), breast cancer, skin cancer, kidney cancer, colorectal cancer, pancreatic cancer and leukaemia. Most of the treatments are currently performed at late-stage disease, meaning metastatic disease.

Side effects

Side effects differ between the treatments and may range from very mild and transient to more severe. Side effects depend on where the target of treatment is. If the treatment targets the thyroid gland, transient symptoms such as pain from the throat are likely. Treatment may necessitate hospitalisation for some days if radiation levels are high. Another example is ^{177}Lu-PSMA therapy, where common side effects are dry mouth, tiredness, nausea and low blood counts.

Because side effects highly depend on the target of treatment and the pharmaceutical used, relevant side effects will always be explained by the treating physician prior to treatment at the hospital, and the patient will be monitored closely by the medical team.

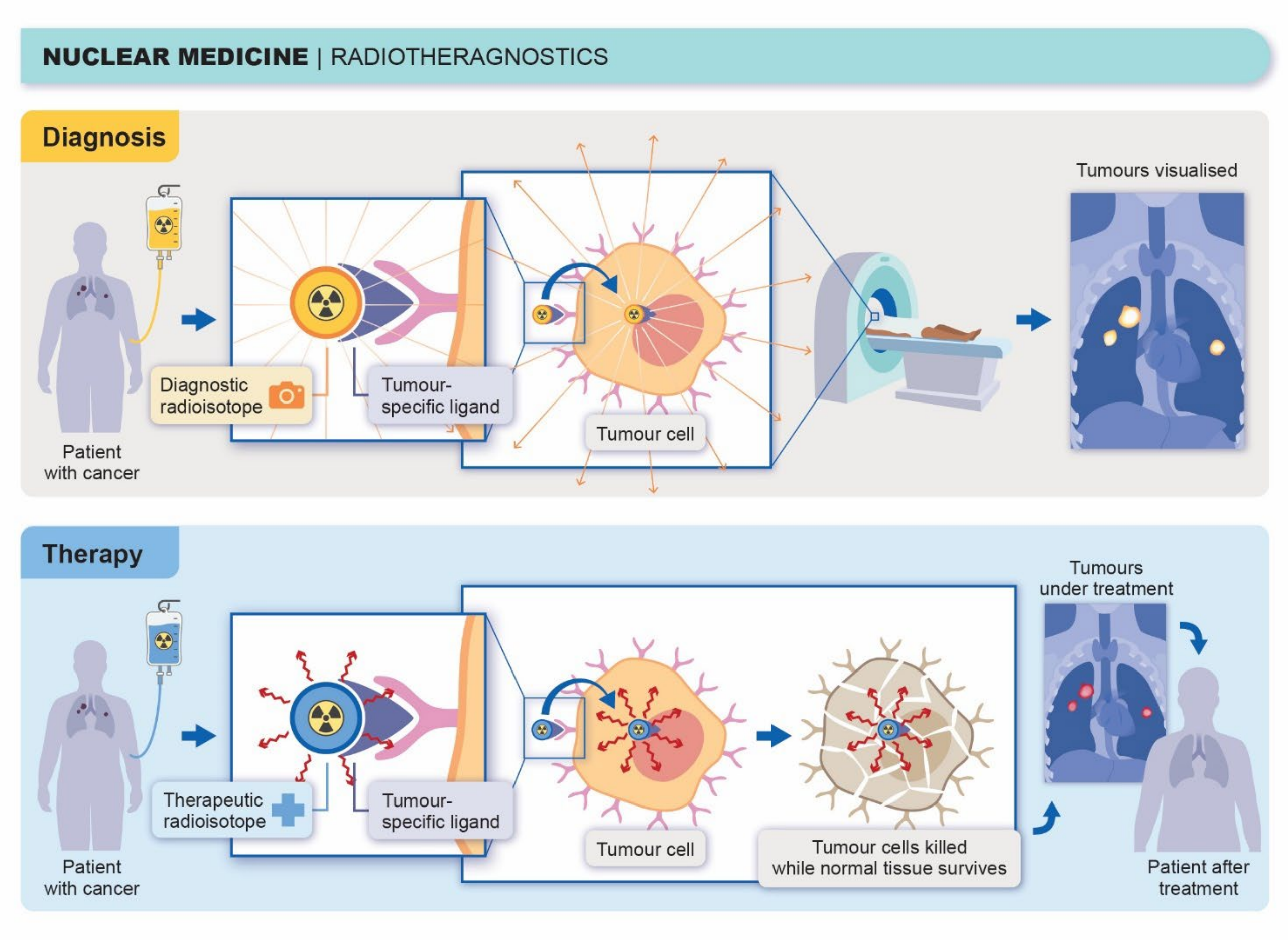

Figure 1: Nuclear medicine therapy employs radioisotopes attached to specific molecules to deliver radiation dose to the tumour. Radiotheranostics is a particular form of nuclear medicine in which the same specific molecule is used for diagnosis and for treatment by changing the attached radioisotope: 1) For diagnosis, a radioisotope that emits photons is employed. 2) For therapy, the radioisotope is electron or alpha particle emitter, causing irradiation and destruction of tumour cells.

Main future forecast

Nuclear medicine therapy is used for treatment of thyroid cancer, neuroendocrine tumours, prostate cancer, hepatocarcinoma, and pancreatic cancer. It is not first line option or standard of care (except

for the thyroid cancer in combination with surgery), but these treatments are advancing in treatment options through the scientific evidence generated in clinical trials.

The approach in which specific molecules seek out and stick to cancer cells is expanding. Instead of targeting only the cancer cells, new treatments are being designed to also target the tumour's surrounding environment. This includes the blood vessels that feed the tumour, supportive cells known as cancer-associated fibroblasts, the structural tissue around the tumour, and even certain immune cells. By targeting these additional components, the tumour may be weakened more effectively.

Another exciting development is the combination of radionuclide treatments with other established cancer therapies, e.g. chemotherapy. Early research and clinical trials are exploring how these targeted radiation treatments might work together with chemotherapy, drugs that make cancer cells more sensitive to e.g. radiotherapy, and immunotherapy. The goal is to enhance treatment effectiveness and improve outcomes for patients.

Overall, these advances represent an important step forward in making cancer treatment more precise and successful.

Useful links

Patient associations or institutions dedicated to improving the access to theranostics:
https://www.oncidiumfoundation.org/patient-support/
https://www.aboutkidshealth.ca/131i-mibg-therapy

Information about clinical trials:
https://www.theranostictrials.org
https://euclinicaltrials.eu/search-for-clinical-trials/?lang=en

Educational material (from radiopharmaceutical and medical devices companies):
PLUVICTO: https://www.pluvicto-hcp.com/psma-positive-mcrpc/resources
LUTATHERA: https://us.lutathera.com/patient-support/useful-resources
TheraSphere: https://www.bostonscientific.com/en-US/medical-specialties/interventional-radiology/interventional-oncology/therasphere/patient-education.html

Sirtex: https://www.sirtex.com/us/patients/resources/educational-materials/

OncoSil: https://www.oncosil.com/resource-library/?resource-category=patient-resources

Radiation protection from the International Atomic Energy Agency: https://www.iaea.org/resources/rpop/patients-and-public/nuclear-medicine

Keywords

Nuclear medicine therapy is known by several names: Radiotheranostics, Targeted Radionuclide Therapy, Theranosis, Radiopharmaceutical Therapy, Molecular Radiotherapy, Radioisotope Therapy, Nuclear Medicine Therapy, or Peptide Receptor Radionuclide Therapy (PRRT), Radioligand Therapy, Radionuclide therapy.

Radionuclides: radioactive isotopes, radioisotopes, imaging radioisotopes, therapeutic radioisotopes

Emitting particles from decays: Alpha particles, beta particles, imaging radioisotopes, therapeutic radioisotopes.

Supplementary S2: Patient information – Radiomics

Radiomics uses computers to measure patterns in medical images (CT, MRI, PET, ultrasound, etc.). Today, it is mainly used in research, but in the future, it may help doctors make more personalised medical decisions.

What is radiomics?

A computer analyses a selected area (e.g. a tumour) on a scan (i.e. a medical image) and measures properties such as shape, texture and brightness patterns. These measurements are called "radiomics features". To put it simply, radiomics means turning medical images into numbers.

Why are researchers studying radiomics?

- To describe a disease more precisely using information already present in medical images.
- To support diagnosis and estimate how a disease may behave over time (prognosis).
- To help choose or adapt treatments and monitor how well a treatment works.

How does radiomics work?

1. You have medical imaging as part of your usual care.
2. A specialist (or dedicated software) marks the area of interest on the image.
3. A computer extracts many measurements (radiomics features).
4. Researchers develop and test a computer model (often using artificial intelligence) on data from many patients.

Is radiomics used in routine care today?

In most hospitals, radiomics is still a research tool. Before it can be used safely in everyday clinical care, radiomics models must be carefully tested in large studies and validated across different scanners, hospitals and patient populations.

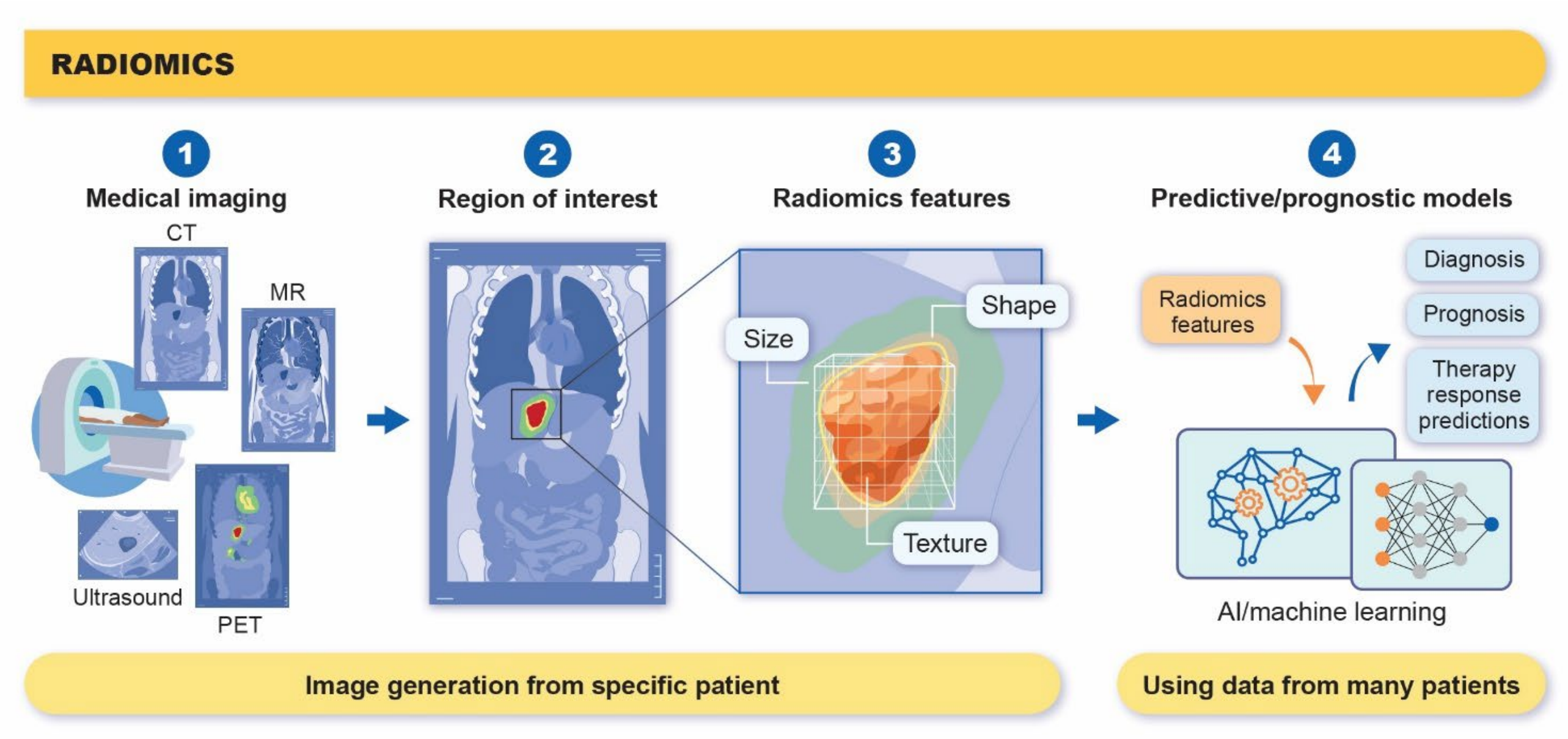

Figure 1. Typical steps in a radiomics analysis workflow: 1) Image/scan acquisition from different medical imaging modalities, either computed tomography (CT), magnetic resonance imaging (MRI), positron emission tomography (PET) or ultrasound. 2) Focus on the region of interest (e.g. the tumour). 3) Extraction of features from the region of interest such as characteristics of size, shape or texture. 4) Integration of numerous radiomic features, extracted from several patients, into AI/machine learning models to make prognosis or predictions.

What could be the benefits?

- More information from your scans, beyond what the human eye can easily see.
- Additional support for doctors when combining information from imaging, pathology and clinical examinations.
- Earlier prediction of how a disease may respond to a treatment.

What are the current limitations?

- Results can change if scan settings or image quality differ.
- A model that works well in one hospital may not perform equally well elsewhere (the model needs to be properly validated to work everywhere).

- Radiomics does not replace your doctors' judgement; it is an additional decision-making support tool.

Your scan and your data

Radiomics usually uses scans that were already performed as part of your care, so it does not need any extra scans. When radiomics is used in research, patient data are handled under strict ethical and legal regulations. Personal identifiers such as your name and hospital number are removed or replaced before your images are examined.

What is needed for the future?

Before radiomics can be widely used in routine care, researchers must ensure that results are reliable and comparable between hospitals. This requires not only large amounts of data, but also shared standards in the way the images are acquired, processed and analysed. International collaboration, data sharing and continuous improvement of methods are key steps toward safe and meaningful clinical use of radiomics.

Questions you can ask your care team

- Is radiomics being used for my condition here, or only in research?
- If it is part of a study, what data will be used and how will it be protected?
- If I do not want my images to be used for radiomics analysis, will there be any consequences on my current treatment?
- Could radiomics results influence my treatment plan today?

Glossary

Artificial intelligence (AI): The ability of computer systems to perform tasks that usually require human intelligence, such as reasoning, decision-making, or problem-solving.

Machine learning: A type of AI where computers learn from examples.

Radiomics features: Numbers that describe an image region (for example shape or texture).

Validation: Testing a model on new, independent data to check that it works reliably.

More information

Cancer Image Europe – Radiomics: https://cancerimage.eu/partners/radiomics

Euro-BioImaging: https://www.eurobioimaging.eu/

Radiomics.bio: https://radiomics.bio/

Open-access review article (PubMed Central): https://pmc.ncbi.nlm.nih.gov/articles/PMC7193906/

Supplementary S3: Patient information – Innovative Radiotherapies

Radiotherapy, or radiation oncology, is a clinical discipline and represents a cornerstone of cancer therapy. It is used alone or in combination with other treatments such as surgery and systemic therapy. Radiotherapy originated over a century ago and continues to evolve. It is widely used across a broad range of cancer types.

What is radiotherapy, and what does "innovation" mean?

Radiotherapy is a cancer treatment that uses ionising radiation to damage the DNA of tumour cells to prevent them from growing and dividing.

"Innovative radiotherapy" refers to modern technologies and clinical workflows that aim to improve the therapeutic ratio, meaning achieving the desired effect on the tumour while reducing side effects in healthy tissues.

Innovation in radiotherapy includes better imaging, more accurate targeting, more sophisticated treatment planning, and improved control of tumour motion, adjusting for movement of the tumour as the patient breathes (e.g. lung tumour). Advanced techniques are now used to track or account for this motion in real-time. This ensures that the radiation is delivered precisely to the tumour while sparing healthy tissues. Furthermore, clinicians now have the ability to adapt plans when anatomy changes, and to ensure rigorous quality assurance to keep treatments safe and reproducible.

In some situations, innovation within radiotherapy has also made treatment more convenient, for example by safely delivering fewer sessions, when clinically appropriate. Breast cancer is one such example, and in many cases treatment is now achieved with just 5-15 sessions (sometimes completed in just one week) instead of the traditional 25–30 daily treatments during a span of 5 weeks.

How does a course of radiotherapy usually work?

Most radiotherapy pathways include a planning phase and a delivery phase. It is delivered over one or more sessions (also called "fractions"). Planning usually begins with a simulation-appointment, where imaging (commonly CT, sometimes combined with MRI or PET) is used to identify the tumour area and nearby organs at risk. Immobilisation devices may be used to help you keep the same position each day. A multidisciplinary team (radiation oncologists, medical physicists, radiation therapists, and

imaging specialists) then creates a treatment plan to deliver the prescribed dose as precisely as possible.

During treatment delivery, accuracy is checked repeatedly. Many innovations are designed to reduce uncertainties: They verify tumour position before each session, account for motion (such as breathing), and in some cases update the plan during the course if the anatomy changes. These steps may not be obvious to patients, but they are central to improving safety and outcomes.

Who may benefit?

Radiotherapy is used across different cancer diagnoses and may be used both to cure cancer and to treat symptoms (e.g. pain) from local tumours.

Innovative radiotherapy approaches are most relevant when increased precision meaningfully changes the balance between tumour control and side effects. Examples include tumours located close to critical organs (where small differences in dose can matter), tumours in areas that can move (where managing movement helps make treatment more accurate), and selected scenarios suitable for stereotactic radiotherapy. Children and young adults may benefit when reducing dose to healthy tissues is particularly important to lower the risk of late effects. Innovation is also important in re-irradiation, when treatment is needed in an area that has already received radiotherapy and dose limits are strict.

Particle therapy may be considered in selected indications where dose reduction to healthy tissues could lower the risk of late toxicity, or where established protocols support its use. Particle therapy is therefore recommended for some patients, but not for all**:** doctors will recommend it when it is expected to offer a clear advantage over standard radiotherapy, taking into account the patient's clinical circumstances.

Side effects

The treatment itself is typically painless. Only the area targeted with radiation therapy is affected, and side effects are therefore specific to the dose and the specific treatment area, as well as treatment type. For example, treatment of tumours in the throat generally leads to more serious side effects including pain in the throat and swallowing difficulties. In contrast, treatment of the breast is associated with milder side effects such as erythema of the skin in the breast area.

Due to variation, relevant side effects will be explained by the treating physician prior to treatment at the hospital.

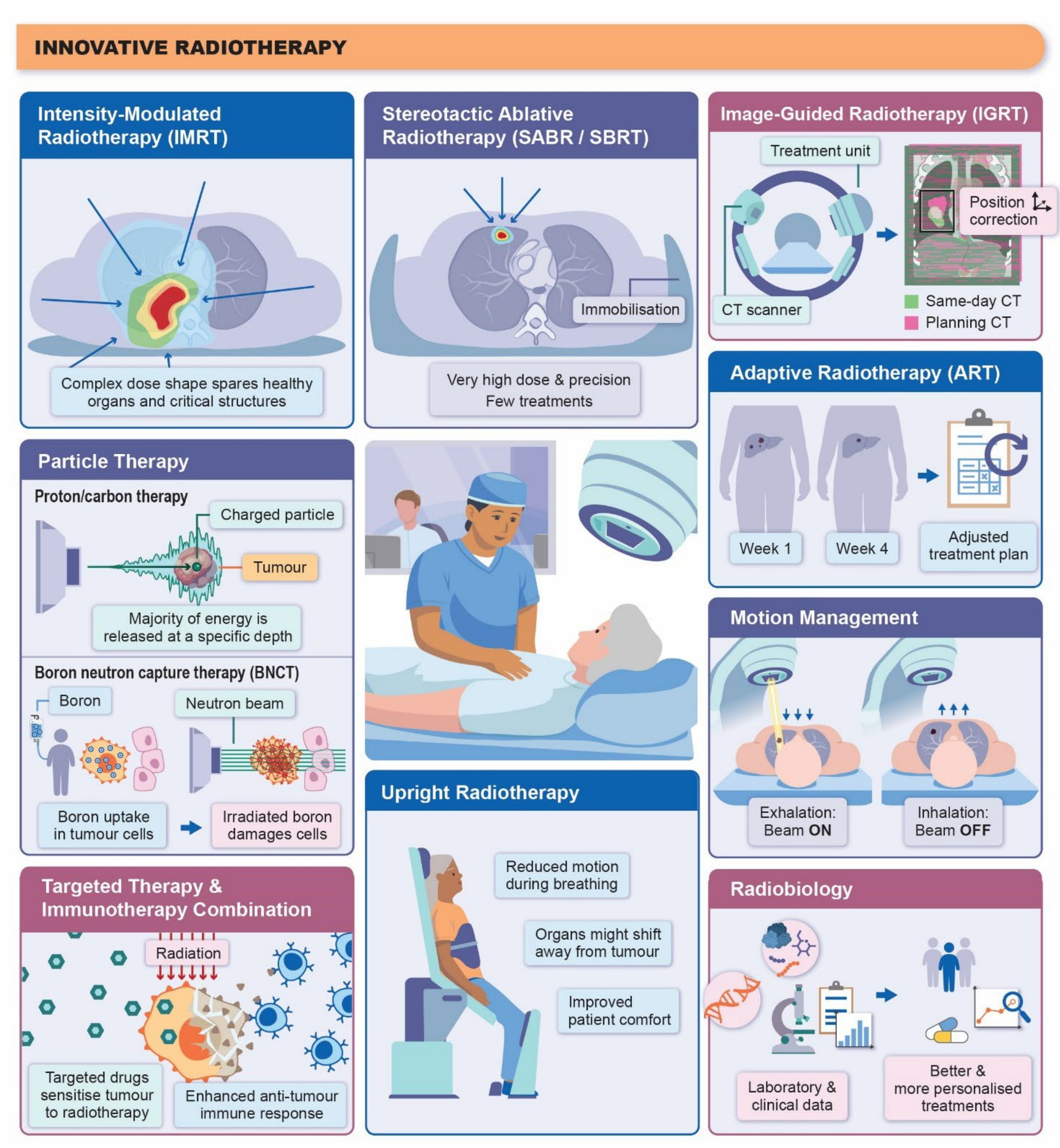

Figure 1: Overview of different types of innovative radiotherapy and related treatment approaches, highlighting how radiotherapy is becoming more precise and personalised, and limiting damage to

healthy tissues. The most suitable technique depends on the type, size and location of the tumour, as well as the aim of treatment.

What does innovative radiotherapy do in practice?

Most patients receive "photon" radiotherapy (high-energy X-rays). Innovative radiotherapy focuses on precision and personalisation. Techniques such as IMRT/VMAT shape and modulate the dose so it conforms closely to the target, helping spare sensitive adjacent organs. Image-guided radiotherapy (IGRT) uses imaging immediately before (and sometimes during) each session to confirm positioning, improving day-to-day accuracy.

A frequent challenge is tumour and organ motion. For cancers in the chest and upper abdomen (for example lung or liver), the target can move with breathing. Motion management approaches, such as 4D imaging, gating, tracking, or surface guidance, help reduce unnecessary "safety margins" by ensuring that the dose is delivered when the tumour is in the expected position. This can improve protection of healthy tissues while maintaining tumour coverage.

In carefully selected cases, stereotactic radiotherapy (SRT/SBRT) delivers very precise, high doses in a small number of sessions (often 1–5). It is used for specific situations such as certain early-stage tumours or limited metastatic disease ("oligometastatic" disease) when clinically appropriate and when normal tissue constraints can be respected.

Another important innovation is adaptive radiotherapy, where the treatment plan can be updated if anatomy changes during the course (for example weight loss, tumour shrinkage, or changes in organ filling). In some centres, MR-guided radiotherapy improves soft-tissue visualisation and may allow plan adaptation during treatment for selected tumour sites.

Particle therapy: Protons and carbon ions

Some centres offer radiotherapy with protons or carbon ions (often referred to as "hadrontherapy"). These particles deposit most of their energy at a specific depth, which may reduce dose beyond the tumour compared with photons. This characteristic can be particularly relevant when the tumour is close to organs where dose should be minimised, and when long-term side effects are a major concern. In this context, one of the main indications for *proton therapy* is in most paediatric solid tumours. For these patients, reducing long-term side effects of treatment is a key goal. Similarly, in certain brain

tumours and head and neck cancers, proton therapy can reduce specific toxicities such as neurocognitive effects in the case of brain tumours, and swallowing difficulties and dry mouth in the case of head and neck cancer.

Carbon ions also have distinct properties, which may be relevant for selected indications under defined protocols (i.e. the base of the skull). Because these treatments require complex, dedicated infrastructure and specialised expertise, they are not available in every country or region.

Emerging therapies (highly specialised): BNCT

Boron neutron capture therapy (BNCT) can be considered an innovative form of radiotherapy, but it is highly specialised and not widely available. BNCT combines a boron-containing drug (intended to accumulate preferentially in tumour cells) with irradiation using neutrons; the interaction can produce very localised tumour cell damage. In most settings, BNCT is offered within clinical trials or tightly governed clinical programmes, and for selected indications based on specific eligibility criteria. Patients interested in BNCT should discuss with their clinical team whether it is relevant for their case and whether referral to a specialised centre is appropriate.

Infrastructure: Who provides it, where is it available, and how can patients access it?

Across Europe, access to innovative radiotherapy is commonly organised in a *two-level system*.

At the first level, many hospital radiotherapy departments and comprehensive cancer centres can provide advanced photon radiotherapy (intensity-modulated radiation therapy (IMRT) and volumetric modulated arc therapy (VMAT)), daily image guidance, and in many cases stereotactic radiotherapy (SBRT). This latter delivers high-dose radiation to small tumours in just 1 to 5 daily sessions and can be performed using standard radiotherapy equipment. For most patients, evaluation and treatment occur within standard oncology referral pathways, close to where they live, and decisions are made by a multidisciplinary team.

At the second level, some technologies are more centralised because they require highly specialised infrastructure. MR-guided radiotherapy is available only at a limited number of centres. Hadrontherapy is delivered in dedicated hubs that typically operate with structured referral criteria and eligibility assessment. According to recent European mapping (2024), approximately 27 clinical proton centres and 4 carbon-ion centres exist; additional proton facilities are planned, but access

remains geographically uneven. This means that the practical question for patients is often whether the most suitable technique can be delivered locally, or whether a referral to a specialised centre is appropriate.

If your team considers that a specialised technology may be relevant, they can initiate a referral for assessment at an appropriate hub. In some situations, this may involve cross-border referral pathways, depending on national rules, reimbursement conditions, and local clinical arrangements.

Important note (availability and eligibility)

Availability of technologies, referral pathways, and eligibility criteria can change over time and may differ between countries. Decisions should always be made with the treating multidisciplinary team, based on clinical appropriateness, expected benefit, and the best available evidence.

Keywords (for patient searching)

IMRT, VMAT, IGRT, SBRT/SRT, motion management, adaptive radiotherapy, MR-guided radiotherapy, proton therapy, carbon-ion therapy, hadrontherapy, BNCT, referral pathways, quality assurance, patient-reported outcomes.

Suggested questions to ask your team

1. Could an advanced technique (such as IMRT/VMAT, SBRT, adaptive or MR-guided radiotherapy) be relevant for my tumour type and location?
2. Is it available locally, or should I be referred to a specialised hub?
3. If particle therapy or an emerging approach is being considered, what evidence supports its use for my situation, and is it offered under a protocol or clinical trial?
4. What are the expected benefits and risks, including potential late effects, and what follow-up is planned?

Useful links for Patients' Associations

Belgium

Stichting tegen Kanker: https://kanker.be

Kom op tegen Kanker: https://www.komoptegenkanker.be

Vlaams Patiëntenplatform vzw (Flanders): https://vlaamspatientenplatform.be

European Cancer Patient Coalition (ECPC): https://ecpc.org

Lithuania

POLA: https://pola.lt/

Italy

AIMaC – Associazione Italiana Malati di Cancro: https://www.aimac.it/

FAVO - Federazione Italiana delle Associazioni di Volontariato in Oncologia: https://www.favo.it/

LILT – Lega Italiana per la Lotta contro i Tumori: https://www.lilt.it/

Europa Donna Italia: https://www.salutedonnaonlus.it/

Europa Uomo Italia: https://www.europauomo.it/

Salute Donna Onlus: https://www.salutedonnaonlus.it/

Norway

Kreftforeningen : https://kreftforeningen.no/

Spain

Asociación Española Contra el Cáncer (clear patient-focused cancer information, psychosocial support and guidance on access to care): https://www.contraelcancer.es

Grupo Español de Pacientes con Cáncer (focused on patient rights, equitable access to innovation and patient advocacy): https://www.gepac.es

Asociación de Madres y Padres de Niñas y Niños con Cáncer de la Comunidad Valenciana (Parents' association supporting children and adolescents with cancer in the Valencian Community)

Inicio – Aspanion:

https://aspanion.es/

Sweden

Cancerfonden (patient-focuse on cancer information, psychosocial support and guidance on access to care):
https://www.cancerfonden.se/

Regionala Cancercentrum I samverkan (focused on cancer information, clinical studies, cancer care guidelines, webinars, workshops and training):
https://www.cancercentrum.se

Nätverket mot cancer (umbrella organisation for cancer profiled patient organisations):
https://www.natverketmotcancer.se

Supplementary S4: Patient information – Innovative Surgery

Making surgery safer and recovery faster

Doctors now focus on preparing patients before, during, and after surgery. This is called enhanced perioperative management.

Before surgery, patients may join prehabilitation programs that include light exercise, good nutrition, and stress-reducing support. Being stronger before surgery helps lower complications and speeds recovery.

Hospitals also use enhanced recovery after surgery (ERAS) routines, which guide pain control, early movement, and when to start eating again. These routines improve recovery, especially in patients with complex abdominal and pelvic cancers.

New digital tools, such as apps for reporting symptoms, may help teams respond earlier if problems arise.

New treatment options before and after surgery

Cancer care is changing rapidly. Immunotherapy and targeted therapies are now standard for many cancers.

More patients receive treatment before surgery (neoadjuvant therapy) to shrink tumours and target potential microscopic spread of cancer tissue. This can make surgery easier, help save organs, or in some cases avoid surgery altogether. It may also render technically inoperable tumours resectable.

Because patients respond differently, surgeons tailor decisions to how well each person's specific cancer reacts. Researchers are developing better biomarkers—tests that help predict response and guide treatment choices.

High-tech tools that make surgery safer and more precise

Robotic surgery and new imaging methods help surgeons clearly see the tumour during surgery.

Tools like fluorescent dyes, real-time scans, and intraoperative MRI help ensure the tumour is removed safely.

In complex surgeries, doctors may use 3D models or augmented views created from a patient's own

scans. These tools improve planning and navigation during surgery. Overall, these technologies reduce uncertainty and support precise cancer removal.

Treatments that target specific areas of the body

These cancer treatments focus on one specific part of the body instead of affecting the whole body. For example, giving very strong doses of chemotherapy directly to an arm, a leg, or an organ. The treatment works powerfully where the cancer is located, while the rest of the body gets fewer side effects.

For cancers in the abdomen or the lining of the abdomen, there are methods where surgery is followed by warm chemotherapy washing the abdominal cavity (HIPEC), or where chemotherapy is sprayed in as a fine mist (PIPAC). New methods explore whether patients can receive chemotherapy directly into the abdominal cavity over time at home through a small catheter placed during surgery.

For cancer that has spread to the liver, treatments that target the liver directly, such as percutaneous hepatic perfusion (PHP) are available. This can help when standard whole-body treatment is not enough.

Gentle, targeted treatments that destroy tumours without major surgery

Some cancers can be treated with ablative techniques, which destroy tumours using focused energy rather than large surgery.

Examples include:

- *HIFU* – (high-intensity focused ultrasound) that heats and destroys tumours.
- *RFA* – (radio frequency ablation) small probe that delivers heat directly into the tumour.

These options are used in organs like the liver and prostate and may preserve organ function and shorten recovery.

Researchers are also exploring how these methods can be combined with major surgery in selected patients.

How artificial intelligence (AI) helps making surgery safer

AI can analyse scans, medical records, and test results to support care before, during, and after surgery.

AI helps with:

- Planning – understanding tumour size and location.
- Guidance – offering real-time support during surgery.
- Recovery prediction – identifying who may need extra follow-up.

AI does not replace doctors but helps them make more informed decisions.

Using big data to personalise cancer treatment

Doctors can now combine information from DNA, proteins, imaging, and laboratory tests - an approach called multi-omics. This helps build a more complete picture of each patient's cancer.

These insights help doctors:

- Understand tumour behaviour.
- Predict treatment response.
- Choose the safest surgical plan.

AI-enhanced tools can also identify which patients may benefit from complex surgery and support robotic surgery through guidance and training.

Where are these treatments available - and how patients can access them?

Advanced surgical technologies are mainly offered in specialised cancer centres and university hospitals. These centres have expert teams, advanced equipment, and access to clinical trials.

Treatment is delivered by multidisciplinary teams of surgeons, oncologists, radiologists, anaesthesiologists, nurses, and rehabilitation specialists.

Patients usually access these treatments through referral to a centre of excellence. Some procedures are available only in a few highly specialised units.

Patients may be referred by their doctor, request a second opinion, or join a clinical trial.

Who might benefit from these advanced techniques?

Many patients with cancer may benefit, especially those who:

- Have locally advanced tumours.
- Have limited metastatic disease.
- Require complex surgery where organ preservation matters.

Innovative surgery methods are used in cancers of the digestive system, gynaecologic organs, urinary system, lungs, chest, and skin, but applications continue to expand across tumour types.

A multidisciplinary team evaluates each case to ensure treatments match the patient's health, cancer type, and preferences.

What can patients expect in the next five years?

Most of these technologies already exist or are being developed in leading centres. Over the next five years, patients may see:

- More personalised treatment: More precise use of scans, blood tests, and digital tools to guide decisions.
- Better diagnostic tests: New biomarkers to detect cancer earlier and track treatment effects.
- Improved access: More collaboration across European countries to ensure equal access to advanced treatments.
- A connected care system: Shared standards, high-quality training, and clearer patient information across Europe.

Useful links

European Cancer Information Portal (EU-CiP) – provides patient-friendly, evidence-based information on cancer diagnostics and treatment options across Europe: https://cancerinfoportal.eu/

Keywords

Innovative surgery; organ-preserving surgery; multimodal cancer treatment; image-guided surgery; regional cancer therapy; ablation; artificial intelligence; precision surgery; ERAS; prehabilitation.

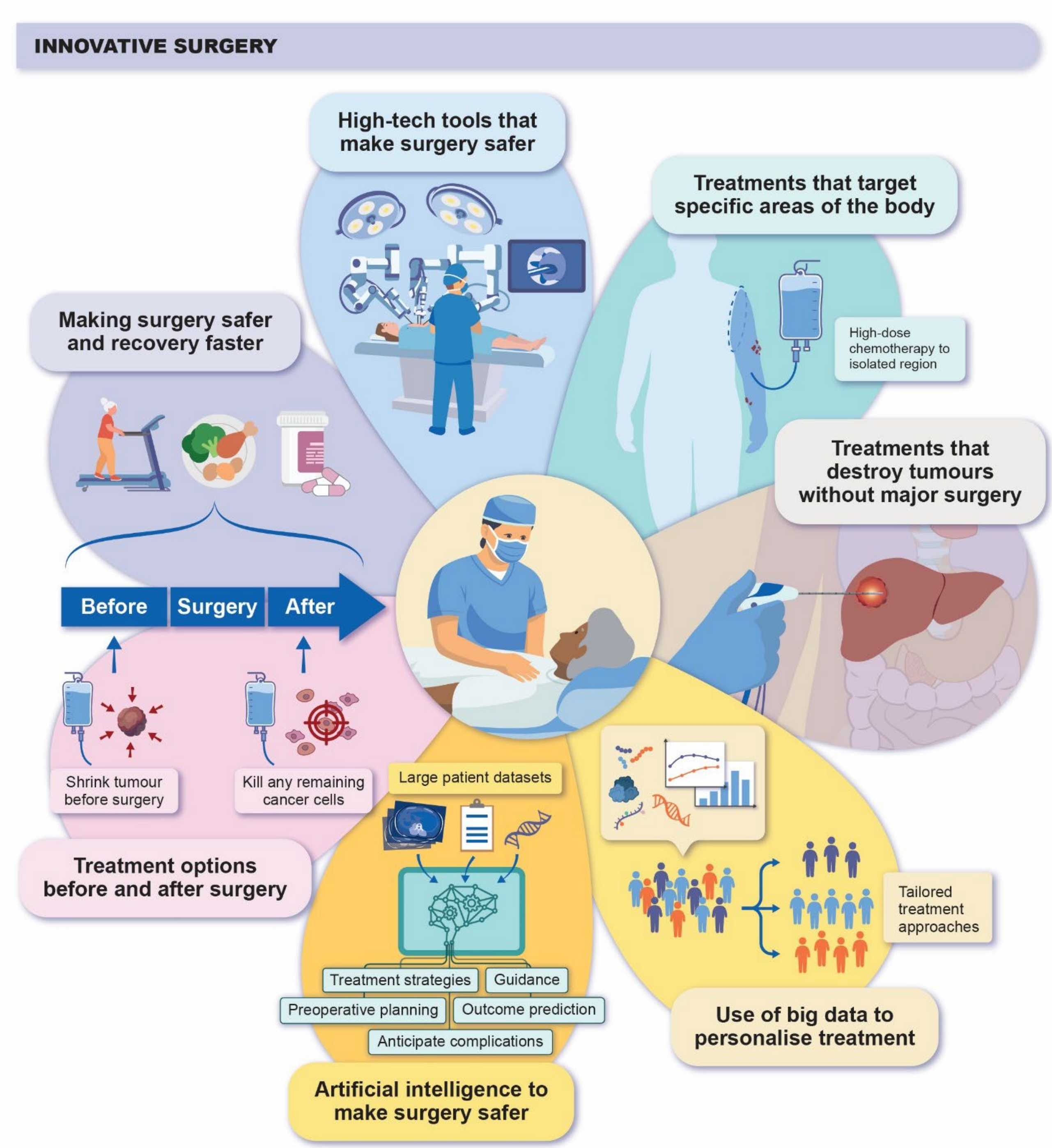

Figure 1: Surgical treatment may apply to all solid cancers. Surgery is often combined with supplementary procedures like medical treatment, radiation or ablation to increase its efficiency and preserve normal tissue and function. Perioperative physiotherapy and nutrition help patients to tolerate the treatment, and imaging platforms help the surgeon to limit the operation to preserve healthy tissue and organs.

Supplementary S5: Patient information – Physical Methods of Ablation

What is ablation?

Physical ablation of tumours is an image-guided treatment, using heating, freezing, electric pulses or ultrasound to achieve complete tumour destruction, usually in one single treatment. Mechanisms of the different ablative techniques are summarised in table 1.

Ablation works across tumour types and can be used when there is one or more tumours that can be safely reached with CT scanning or ultrasound image guidance.

Other medical treatments can generally be given alongside tumour ablation.

Procedure

Tumour ablation is performed by interventional radiologists, surgeons, oncologists or dermatologists, depending on the location of the tumour and the required ablation technique.

Ablation is achieved by inserting one or more thin needles in the tumour (minimally invasive procedure) or using focused ultrasound from outside the body (non-invasive procedure). In either case, some form of anaesthesia is required, either local anaesthesia, conscious sedation or general anaesthesia. Ablation is usually performed at a hospital as an outpatient procedure and is usually performed with a single treatment.

Who may benefit?

Ablation is an approved treatment which can be used for both curative and palliative (alleviation of the symptoms) purposes and for various types of cancers. It may be considered when:

- Only one or more tumours are present, are of suitable size, and it is physically possible to treat them using an ablation technique.
- Metastatic lesions are causing symptoms such as pain or bleeding, which can be reduced by tumour ablation.

Examples of use of ablation:

- Metastases to the liver: Radiofrequency or microwave ablation, electroporation.
- Metastases to the skin: Electrochemotherapy.

- Metastases to lymph nodes: Cryotherapy.

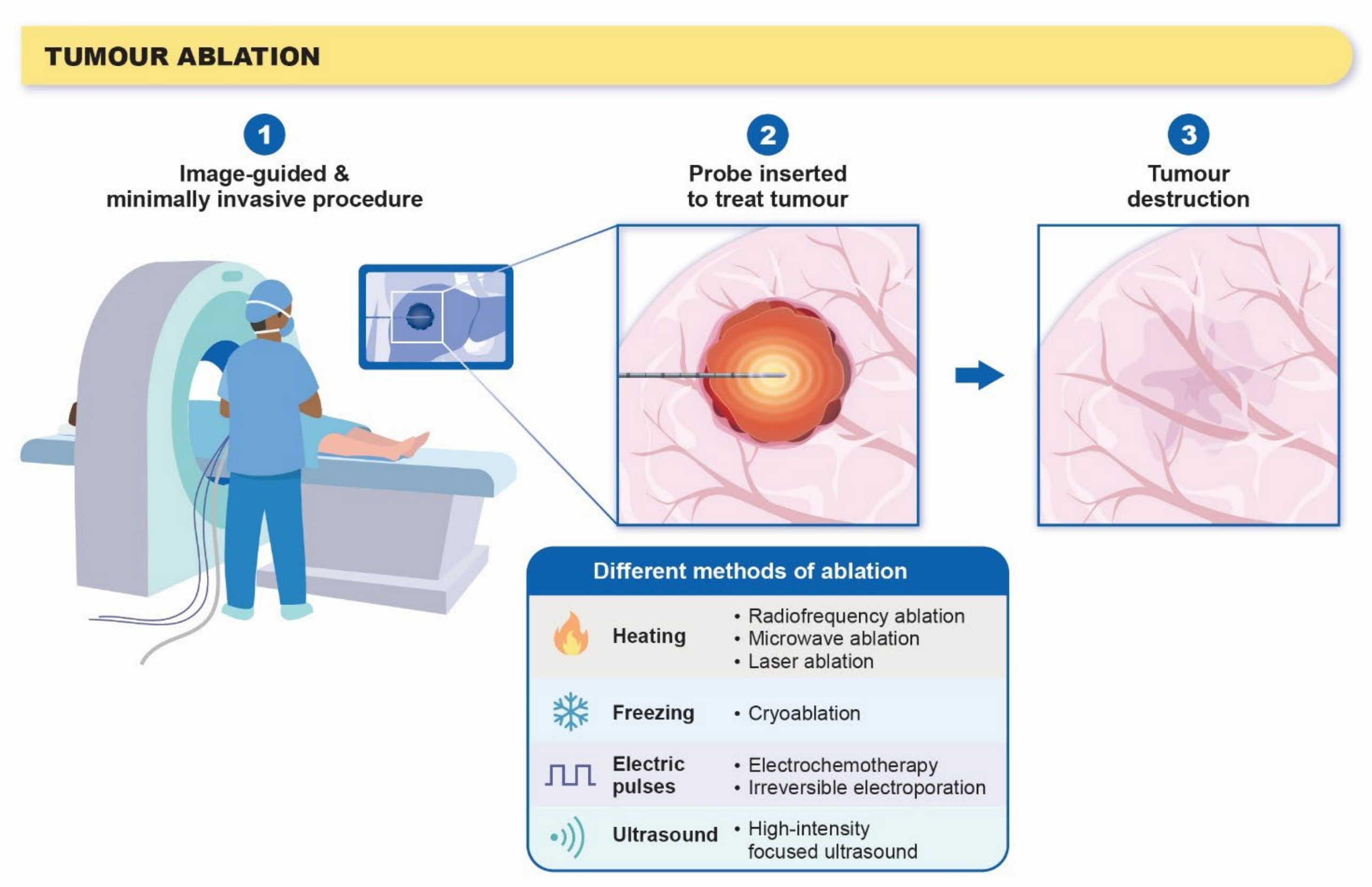

Figure 1: Ablation methods work across cancer types, exemplified here with a liver tumour and microwave ablation. The tumour is first visualised, and a thin needle (probe) is then inserted into the tumour and turned on. Tumour is destroyed, typically with one single treatment.

Success rate

Ablation of tumours is highly effective, with an estimated success rate of 75-95% complete tumour eradication.

Side effects

- <u>Insertion of electrodes:</u>

  Pain, transient and restricted to treatment site (common side effect).

  Bleeding and infection which is a risk with any invasive procedure (rare side effects)

- Anaesthesia

  Anaesthesia rarely causes complications.
- Other side effects

  Other side effects specific to the individual procedure will be addressed by the treating physician.

Advantages

- Minimally invasive or non-invasive procedure.
- Tumour destruction is typically achieved with just one treatment.
- Local treatment which does not involve major surgical procedures (less complications/side effects compared to surgical resection).
- Quick recovery.
- Admitted and discharged the same day.

How and where to get it

Decisions concerning ablative treatment options are usually made at multidisciplinary team meetings where specialists from e.g. oncology, surgery and radiology meet about patient cases.

Ablation procedures are performed in larger hospitals or specialised cancer centres by highly trained specialists. If your local hospital does not perform tumour ablation, referral to another hospital is possible.

Approved ablative techniques

Ablation technique	How it works
**Radiofrequency ablation**	A probe is placed in the tumour, and heat is generated using radiofrequency waves. The heat gradually spreads through the tumour and destroys the cancer cells.
**Microwave ablation**	A heat generating technique similar to radiofrequency ablation but using a probe with microwaves.

**High intensity focused ultrasound**	Ultrasound waves are directed towards the tumour causing cancer cells to be destroyed by heat. This can be done by placing the ultrasound probe on the skin or placing the probe inside the body (for example in the pee tube near the prostate).
**Cryotherapy**	In cryotherapy one or more probes are inserted into the tumour. The extreme cold freezes and destroys the cancer cells.
**Laser ablation**	Laser ablation is a thermal ablation method that works by placing light fibres in the tumour and by heating the tumour.
**Irreversible electroporation (IRE)**	Electroporation is not a thermal method. It uses short electrical pulses to damage and destroy cancer cells without heat. Electroporation is performed using one or more needles inserted into the tumour.
**Electrochemotherapy (ECT)**	In electrochemotherapy, electroporation is performed at lower electrical pulse strengths and is combined with a chemotherapeutic agent, making the chemotherapy more effective in the treated area.

Table 1: Ablation techniques and mode of action

Future perspectives

Although several approved methods exist for tumour ablation, research within this field is constantly progressing, and ablation therapies are expected to further improve and expand. This includes both development of ablation probes for larger tumours, new computer-based guidance systems for more accurate insertion of needle(s), and use of radiological imaging to improve the effectiveness.

As medical treatment is also advancing, more patients are likely to benefit from the *combined* effect of medical therapy and ablation. Systematic integration of ablation techniques into clinical guidelines is gradually advancing (2026), raising awareness of tumour ablation options and strategies and support shared decision making within cancer treatment and palliation.

Useful links

Cardiovascular and Interventional Radiological Society of Europe (providing information about ablation and other interventional radiology procedures): https://www.cirse.org/patients/general-information/ir-procedures/

Keywords

Radiofrequency ablation (RFA), microwave ablation (MWA), high intensity focused ultrasound (HIFU), cryotherapy (Cryo), laser ablation, irreversible electroporation (IRE), electrochemotherapy (ECT), tumour ablation, physical methods of ablation

Supplementary S6: Patient information – Cell Therapies

Cell therapies are part of what is called *advanced therapy medicinal products (ATMPs)*, a group of innovative treatments that use living cells to treat complex diseases. These cells can be taken from the patients themselves or from a healthy donor. In the lab, they can be modified—or "engineered" when genetically changed—to perform specific therapeutic tasks, such as recognising and destroying diseased cells.
A well-known example is *CAR-T cell therapy*, where certain immune cells are engineered to specifically target and attack cancer cells.

What is the procedure?
For cell therapies, the cells needed for treatment are collected in a procedure called *leukapheresis*. During this procedure, blood is taken from one arm, the specific cells are separated, and the rest of the blood is immediately returned to the body. This procedure is moderately invasive but well-established, performed by trained professionals, and considered safe, as it has been used for many years in transfusion medicine. In some cases, cells may be collected from bone marrow or through a tissue biopsy.

Generally, patients can expect to leave the hospital on the same day, as the collection is typically performed as an *outpatient procedure*.

Once collected, the cells are sent to a specialised laboratory where they are grown, modified, or genetically engineered under strictly controlled conditions. This preparation process usually takes about 4-6 weeks before the cells are ready to be re-infused.

After thorough quality and safety checks, the modified cells are returned to the patient via a simple infusion, similar to a blood transfusion. The standard treatment plan normally involves one cycle of therapy. Sometimes, a preparatory treatment, such as a short course of medication, is needed to help the modified cells work effectively once they are infused back into the body. These treatments represent an important step toward *precision medicine*, which means personalised care that takes into account each patient's unique biological and immune characteristics.

What are the side effects?

Like all advanced therapies, cell therapies can cause *side effects*. Their severity varies from person to person and depends on the type of therapy, the patients' overall condition, and how their immune system responds. For this reason, patients are monitored closely by highly specialised teams who can quickly recognise and manage any complications.

Systemic effects (whole body):

The most common reaction is cytokine release syndrome (CRS). This feels like a severe flu, with fever, fatigue, or low blood pressure. While it can be intense, it is usually transient (temporary), appearing within a few days of the infusion and resolving within a week or two with hospital care. Some patients may also experience neurological effects, such as temporary confusion or difficulty speaking (ICANS). These symptoms are also generally transient and disappear as the body stabilizes. Finally, because the treatment targets certain cells, you might experience a drop in healthy blood cells (Cytopenia), which can last for a few weeks, or a long-term reduction in B-cells (B-cell Aplasia). The latter may persist as long as the therapy is active in your body, but it can be managed with routine medical support.

Local effects (site-specific)

If your cells are collected via a tissue biopsy or bone marrow aspiration, you may feel some local soreness, bruising, or discomfort at the site of the procedure. These effects are mild and transient, usually fading completely within a few days. Similarly, the site where the modified cells are infused might show minor redness or swelling, which typically clears up within 24 to 48 hours.

Summary of duration

Most side effects are short-term and occur while you are under close medical supervision. While some immune-related changes can persist longer, your medical team will provide ongoing monitoring to ensure your safety and quality of life throughout the journey.

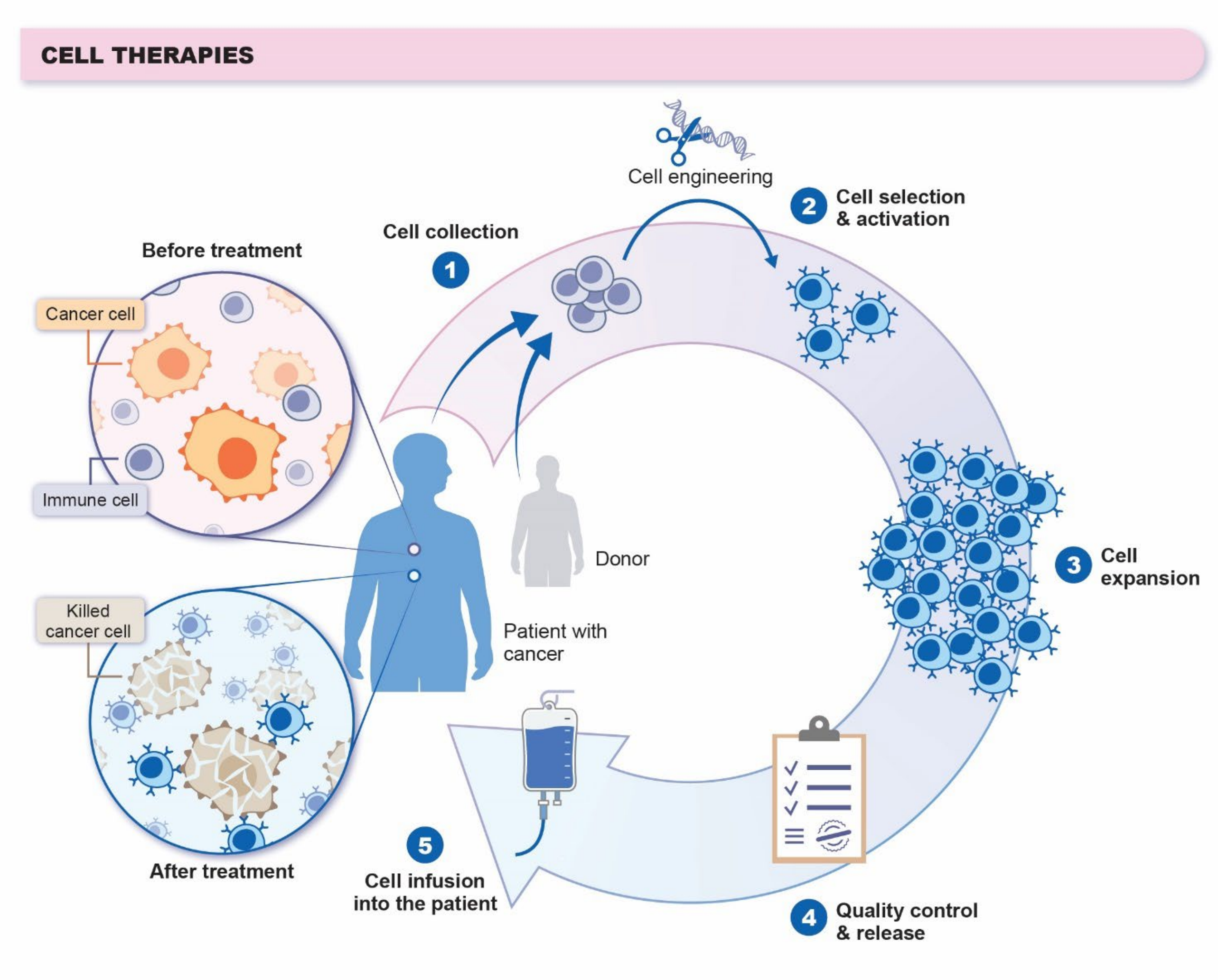

Figure 1: The concept of cell therapies. 1) Cells are collected from healthy donors (allogenic) or from patient (autologous). 2) Target immune cells are isolated, activated with specific stimuli and in some cases genetically engineered to enhance their therapeutic function, including CAR insertion. 3) The cells are grown and increased in number in a laboratory before being used for treatment. 4) The cell product undergoes extensive safety and quality testing. 5) The final product may be infused to the patient.

Who performs it and where can I get it?

Because of their complexity, cell therapies are administered only in *highly specialised and accredited hospitals.* These centres have the advanced skills and dedicated facilities required for the treatment. The care process involves several professionals, including:

- Doctors specialised in advanced therapies, who assess if it is possible to treat, and oversee the treatment journey.
- Laboratory biologists and technicians, who prepare the cells in controlled and safe environments.
- Nurses and clinical staff, who assist patients, monitor side effects, and ensure continuous care.

Treatment usually takes place in a *hospital setting* and often in a centralised location connected to a network of reference centres that refer patients and share common protocols. Not all oncology or haematology centres can administer these therapies, so collaboration between facilities is essential. In some cases, treatment may be carried out abroad, although cross-border access can be complicated due to different rules regarding reimbursement, authorisation, and follow-up. For more information about treatment and referral options, you should contact your treating physician.

Who may benefit?

Cell therapies are already approved for several *blood cancers*, such as leukaemia, lymphoma, and multiple myeloma, as well as for some *solid tumours* like melanoma and synovial sarcoma. For many other cancers - breast, lung, prostate, gastrointestinal, head and neck, urogenital - research is still ongoing, with clinical trials assessing safety and effectiveness. In the future, these therapies may also be used outside oncology, for certain *autoimmune diseases*, and will likely become available for an increasing number of cancers as research progresses.

What are the future perspectives?

The goal of new cell therapies is to be more *effective, safer, easier to administer, and accessible* to more patients. At the European level, initiatives are underway to improve regulation, access, and patient information, including the creation of official lists of accredited treatment centres. Some therapies that are currently experimental may become part of *standard clinical practice*, offering new options to patients who currently have limited alternatives.

Glossary

ATMP – Advanced Therapy Medicinal Products
European category that includes advanced therapies based on cells, genes, or tissues. These treatments are highly innovative and strictly regulated.

CAR-T
Short for *Chimeric Antigen Receptor T cells*. These are immune cells from the patient that are modified in the laboratory to recognise and attack cancer cells.
The engineering process involves adding a gene called the Chimeric Antigen Receptor (CAR) to T cells. This new gene helps the T cells detect and fight tumour cells. In other words, CAR-T cells are specially designed to find and attack cancer cells in a targeted way.

Precision Medicine
An approach that tailors treatment to the specific characteristics of each patient (genetic, biological, or immunological).

Tissue Biopsy
A procedure in which a doctor removes a small sample of tissue from the body. This can be done with a needle, a small surgical instrument, or under imaging guidance like ultrasound or CT.

Leukapheresis
(A combination of Greek words meaning "white" and "to remove") – a process that removes white blood cells (leukocytes) from the blood.

Autoimmune Diseases
Chronic conditions in which the immune system mistakenly attacks healthy tissues, caused by a combination of genetic and environmental factors.

Useful links

The European Medicines Agency (for simple explanation of what advanced therapies (like gene and cell therapies) are and how they are checked for safety, quality, and effectiveness in the EU:
https://www.ema.europa.eu/en/human-regulatory-overview/advanced-therapy-medicinal-products-overview

The Spanish Agency for Medicines and Health Products (information about cell therapies): https://www.aemps.gob.es/medicamentos-de-uso-humano/medicamentos-biologicos/terapias-avanzadas/

Supplementary S7: Patient information – Ex-vivo Testing of Agents

Why test cancer cells outside the body?

Cancer treatments are often chosen using standard guidelines and genetic tests. While this works for many people, it does not always show which treatment will be most effective for an individual patient. Cancers that seem similar can have significant differences, resulting in very different responses to treatments. *Ex-vivo* testing offers a complementary approach. It allows researchers and clinicians to study how a patient's own cancer cells respond to different treatments/drugs *outside the body*, in a laboratory. This helps move cancer care toward more personalised treatment decisions.

What is ex-vivo testing?

Today, biopsies are already part of routine cancer care, mainly used to *diagnose the cancer* and to analyse its *genetic characteristics*. With *ex-vivo* testing, the procedure to collect the sample is usually the same or very similar and it can also be obtained during surgical tumour removal. Researchers take a small portion of the patient's tumour and keep the cancer cells alive in the laboratory. These cells are then exposed to different treatments, allowing scientists to observe and study which therapies are most effective for that individual tumour.

How can ex-vivo testing help guide cancer treatment?

The main goal of *ex-vivo* testing is to *support more personalised treatment*, moving away from a "one-size-fits-all" approach and toward treatments that are more closely adapted to each individual patient. Traditional cancer treatments-such as surgery, chemotherapy, radiotherapy, and hormonal therapy- do not always fully account for differences between patients or the complexity and variability of individual tumour. Thus, *ex-vivo* testing looks at *how your own cancer cells respond to different drugs*. By observing which treatments seem effective — and which do not — doctors and researchers hope to better *guide treatment choices*, especially when several options are possible. The long-term aim is to save time and increase efficiency by avoiding useless therapies.

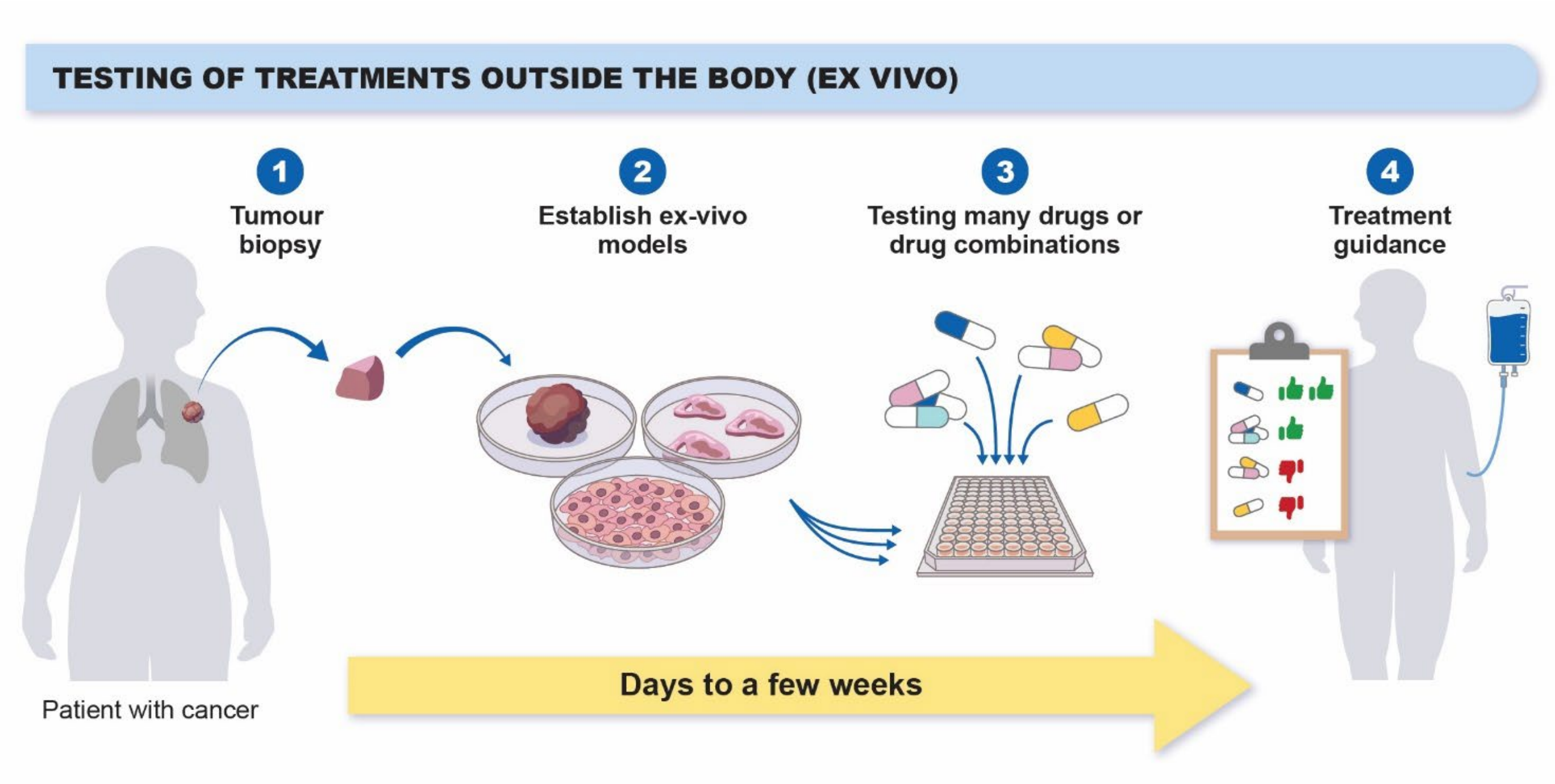

Figure 1: An overview of ex-vivo drug testing and how it may be integrated into clinical care. A patient's tumour sample is collected at diagnosis and/or at relapse as part of routine clinical procedures. Tumour cells are then processed in the laboratory using an ex-vivo drug sensitivity screening platform. Multiple drugs are tested in parallel, which makes it possible to evaluate potential treatments. The results are then discussed by a multidisciplinary molecular tumour board, often together with genomic profiling data, to help inform individualised treatment decisions.

Is ex-vivo testing already part of routine cancer care? How does the process work?

At the moment, *ex-vivo* testing is *not yet part of routine clinical care*. It is still conducted within *research settings* and clinical studies. This means the methods are evolving and being carefully evaluated. After the tumour sample is collected, it is sent to a laboratory. There, the cancer cells are *processed and grown under controlled conditions.* Testing drugs on living cells also requires days or weeks of careful work.

What types of ex-vivo technologies are currently being studied?

Several *ex-vivo* technologies are being evaluated today. All of them aim to study how cancer cells respond to treatment outside the body, but they do so in different ways:

- *Patient-derived cancer cell lines*: Tumour cells grown in flat (2D) cultures.
- *tumour explants/tumour tissue slices:* Thin sections of fresh tumour tissue kept alive for short-term testing.
- *Patient-derived organoids/assembloids*: Three-dimensional "mini-tumours" grown in the laboratory. When other cells, e.g. tumour microenvironment cells, are added to organoids, another form of advanced "mini-tumour" is developed, called assembloid.
- *Patient-derived xenografts (PDX):* Tumour tissue implanted into mice to preserve tumour complexity.
- *Tumour-on-chip systems*: Micro-devices that recreate key aspects of the tumour environment.

Some technologies may prove more useful than others, and new approaches may be developed in the future as science progresses.

What benefits could it bring to patients?

If successfully integrated into care, *ex-vivo* testing could bring several important benefits: *More personalised treatments*, better adapted to each patient's tumour; *Alternative therapeutic options*, especially in complex situations or when standard treatments no longer work; *Drug repositioning*, meaning existing medicines could be identified as useful for a different cancer than originally intended

By identifying treatments that are unlikely to work, this approach may also help *avoid wasting time on inappropriate treatments, with all their drawbacks* and focus on options with a higher chance of benefit.

What are the limitations of ex-vivo testing?

Since *ex-vivo* testing is still in the research phase, there is an important *element of uncertainty*. Sometimes cancer cells cannot be grown successfully in the laboratory, or the results may not fully reflect what happens in the patient's body. There are also scientific challenges, such as reproducing the complexity of the immune system or maintaining stable cell cultures over time. For these

reasons, *ex-vivo* testing can sometimes *fail or provide false positive, negative or inconclusive results*, and it cannot yet replace standard clinical decision-making. However, compared with other out-of-body model systems currently available, ex-vivo approaches more closely reflect the patient's own tumour biology. Ex-vivo testing is not infallible-but neither are current standards of care. In fact, the limitations of existing treatments are precisely what have driven the development of ex-vivo systems. Being transparent about these limits is essential so that patients understand both the *promise and the current boundaries* of this approach.

Keywords: Functional precision oncology; Patient-derived cells; Patient-derived organoids; Tumour tissue slices; Tumour-on-chip systems; Drug Screening

Useful links:

Short educational videos:

Understanding ex-vivo testing and personalised cancer treatment:
https://www.youtube.com/watch?v=E_GpS410OpQ

Functional precision oncology; how treatments are tested on tumour cells:
https://www.youtube.com/watch?v=ydHmkxZ2jIQ

How cancer cells are tested outside the body:
https://www.youtube.com/watch?v=vY9Ij5uZTgc

Personalised approaches in cancer care:
https://www.youtube.com/watch?v=lBlFwuEQW_E

What is a PDX mouse model?:
https://www.youtube.com/watch?v=nYbR7BHY_Dw

2D and 3D cancer models and organoids; Organoid technologies in research:
https://www.youtube.com/watch?v=vywZVtDyj9E

Further sources of information:

Wikipedia (functional drug sensitivity testing, general overview): https://en.wikipedia.org/wiki/Functional_drug_sensitivity_testing

Scientific publication "Precision oncology using ex vivo technology: a step towards individualised cancer care?": https://pmc.ncbi.nlm.nih.gov/articles/PMC9884776/

Mayo Clinic (example of an ex-vivo research study): https://www.mayo.edu/research/centers-programs/center-individualized-medicine/research/clinical-studies/ex-vivo

Review "Ex vivo drug screening: an emerging paradigm in the treatment of childhood cancer" (provides information about ex vivo drug screening and how it can be performed): https://eprints.whiterose.ac.uk/id/eprint/225452/8/Ex_vivo_drug_screening_pediatric_v1.4_DK.pdf
